# Context-dependent functions of the aryl hydrocarbon receptor in gastrointestinal cancers: from microenvironmental regulation to precision targeted therapy

**DOI:** 10.3389/fcell.2026.1886657

**Published:** 2026-08-20

**Authors:** Jiao Ma, Shenghao Li, Yining Qiao, Zihan Gao, Jinzhe Liu, Yanru Song, Yu Liu, Xiaohui Ji, Jianbo Li, Jie Zhang, Liang Chang, Bingjie Huo

**Affiliations:** 1 Oncology Department of Integrated Chinese and Western Medicine, The Fourth Hospital of Hebei Medical University, Shijiazhuang, Hebei, China; 2 Key Laboratory for TCM Diagnosis and Treatment of Digestive Tract Tumors in Hebei Province, The Fourth Hospital of Hebei Medical University, Shijiazhuang, Hebei, China; 3 Department of Basic Theory of Traditional Chinese Medicine, Hebei University of Chinese Medicine, Shijiazhuang, Hebei, China

**Keywords:** aryl hydrocarbon receptor, gastrointestinal cancer, signaling pathways, targeted therapy, tumor microenvironment

## Abstract

The aryl hydrocarbon receptor (AhR) is a ligand-activated transcription factor with context-dependent roles in gastrointestinal (GI) tumor development. Depending on cellular context and microenvironment, AhR can preserve epithelial integrity, suppress inflammation, and inhibit tumor growth, but it can also promote immune evasion and metabolic reprogramming to drive tumor progression. Most existing reviews have focused on a single GI tumor type or functional dimension, and the concept of AhR as a context-dependent signaling hub has not been effectively linked to therapeutic stratification across the full spectrum of GI malignancies. No prior review has systematically compared AhR across five GI cancer types—esophageal, gastric, colorectal, hepatocellular, and pancreatic—or addressed the translational gap between preclinical data and clinical application. This review addresses these gaps in three key ways. First, it provides the first head-to-head comparative analysis of AhR functions across these five cancer types. Second, it adopts a functional stratification framework integrating five core mechanistic dimensions—tumor stemness, epithelial-mesenchymal transition, immune remodeling, metabolic reprogramming, and drug resistance—and proposes a three-dimensional AhR stratification model. Third, it systematically discusses emerging AhR-targeted therapeutic strategies—including antagonists, selective AhR modulators (SAhRMs), PROTACs, combination therapies, and microbiome-based interventions—while critically evaluating translational challenges. By establishing this context-informed framework, we aim to provide a conceptual basis for biomarker-guided evaluation of AhR-targeted strategies in GI cancers.

## 1 Introduction

Gastrointestinal cancers remain a leading cause of cancer-related mortality worldwide (Park et al., 2025). Their development is a multi-step process driven by intricate interactions between genetic, epigenetic, and environmental factors (Xu et al., 2025). Among these, environmental influences—particularly dietary chemical pollutants and microbial metabolites—play critical roles in tumor initiation and progression (Rajabi et al., 2022). For instance, polycyclic aromatic hydrocarbons (PAHs) contribute to carcinogenesis at least in part through activation of the aryl hydrocarbon receptor (AhR). As a key link between environmental signals and cellular responses, AhR is not only involved in classical xenobiotic metabolism but also regulates cell cycle progression, immune responses, and energy metabolism (Coumoul et al., 2026). Within the gastrointestinal tumor microenvironment, AhR activity is tightly modulated by a diverse array of ligands, including environmental pollutants, dietary indole derivatives, endogenous tryptophan metabolites, and products of the gut microbiota (Ma et al., 2026).

Accumulating evidence indicates that AhR is aberrantly expressed in gastrointestinal tumors, where it exerts context-dependent dual functions—either promoting or suppressing carcinogenesis—through cross-talk with key signaling networks, including the Wnt/β-catenin, KRAS, and p53 pathways (Takei and Shimizu, 2026). For example, in hepatocellular carcinoma, kynurenine activates AhR to drive proliferation and induce immunosuppression (Wu et al., 2021). In colorectal cancer, the environmental endocrine disruptor nonylphenol promotes tumor angiogenesis and growth by activating the AhR/HDAC2/HNF4α axis (Zhang T. et al., 2022). Conversely, the phytochemical p-hydroxycinnamic acid inhibits the growth of HepG2 hepatocellular carcinoma cells and induces apoptosis through AhR activation (Yamaguchi et al., 2021). This functional duality renders AhR a highly promising yet challenging therapeutic target (Kazzaz et al., 2024).

The development of AhR-targeted drugs is progressively shifting from toxicology toward oncology. On one hand, antagonists such as IK-175 can improve the tumor immune microenvironment and exhibit marked anti-tumor activity when combined with anti-PD-1 antibodies (Mcgovern et al., 2022); IK-175 has recently completed a first-in-human phase 1/1b clinical trial (NCT04200963) in advanced solid tumors and urothelial carcinoma, demonstrating favorable safety, confirmed target engagement, and preliminary clinical activity when combined with nivolumab (Aggen et al., 2026) (detailed in Section 4.1). On the other hand, agonists like indole-3-carbinol (I3C) can suppress tumor growth through AhR activation in preclinical settings (Liu et al., 2021), though clinical evidence for I3C as a cancer therapeutic remains limited. Recent preclinical evidence further demonstrates that diet-derived AhR ligands, including I3C, are essential for the optimal efficacy of anti-PD-1 therapy: dietary AhR ligand deprivation significantly impairs checkpoint blockade response, while I3C supplementation restores anti-PD-1-mediated reinvigoration of progenitor exhausted CD8 T cells (De Juan et al., 2025). However, the remarkable complexity of AhR signaling, its pronounced tissue specificity, and its dual functional effects pose substantial challenges for drug development (Zhang W. et al., 2022). Notably, sorafenib—a potent AhR antagonist—exerts its anti-hepatocellular carcinoma effects partly via inhibition of the AhR pathway, but may also induce AhR-related endocrine disruption (Wei et al., 2022). Thus, a comprehensive understanding of AhR’s precise mechanisms of action within the distinct microenvironments of various gastrointestinal tumors is essential for unlocking its therapeutic potential.

Although several comprehensive reviews have addressed the role of AhR in cancer, the majority have concentrated on a single tumor type or a singular functional dimension, and the emerging concept of AhR as a context-dependent signaling hub has not been effectively bridged with therapeutic stratification strategies. The present review distinguishes itself from prior works in three key respects: it provides the first comparative analysis across five major gastrointestinal cancer types; it adopts a functional stratification perspective integrating five core mechanistic dimensions (tumor stemness, epithelial-mesenchymal transition, immune microenvironment remodeling, metabolic reprogramming, and drug resistance); and it offers the first systematic discussion of emerging AhR-targeted modalities—including SAhRMs and PROTAC-based degraders—in the context of gastrointestinal malignancies. To translate the mechanistic complexity of AhR into clinically actionable strategies, we employ a three-tiered framework—progressing from mechanistic dissection through functional stratification to translational application—under which we critically evaluate current therapeutic strategies and identify key barriers to clinical translation.

To facilitate consistent analysis across cancer types, we herein operationally define two functional modes of AhR in gastrointestinal oncology. The “gatekeeper” mode refers to AhR activity that preserves epithelial barrier integrity, maintains immune homeostasis, regulates stem cell differentiation, and suppresses inflammation-associated tumorigenesis—typically driven by transient, physiological ligands such as dietary indoles (I3C, DIM) and microbiota-derived tryptophan metabolites (IAA, IPA) in non-malignant or early-stage tissues (De Juan et al., 2025; Ye et al., 2022; Ran et al., 2026). This concept extends the original characterization of intestinal epithelial cells as “gatekeepers” for AhR ligand availability to encompass the receptor itself as a gatekeeper of mucosal homeostasis (Schiering et al., 2017). Conversely, the “promoter” mode denotes AhR activity that drives proliferation, immune evasion, metabolic reprogramming, epithelial–mesenchymal transition, or drug resistance—typically sustained by persistent environmental agonists (e.g., TCDD, PAHs) or tumor-derived kynurenine via the IDO1/TDO2 pathway in established tumors (Wu et al., 2021; Ala, 2021; Anderson, 2020; Anderson, 2022). The transition between these modes is not binary but context-dependent, governed by ligand identity, disease stage, microenvironmental composition, and cell-type specificity; accordingly, the same AhR-targeted intervention may yield diametrically opposite outcomes depending on which mode predominates. Throughout this review, we apply these terms consistently across all five GI cancer types to enable cross-cancer comparative analysis and to anchor the functional stratification framework proposed.

## 2 Structure of AhR and canonical/non-canonical signaling pathways

### 2.1 AhR protein structure and ligand-binding properties

AhR is a member of the basic helix-loop-helix/Per-ARNT-Sim (bHLH/PAS) protein family and comprises three major functional domains: an N-terminal bHLH domain responsible for DNA binding and dimerization, a central PAS domain (consisting of PAS-A and PAS-B, with PAS-B serving as the ligand-binding pocket), and a C-terminal transactivation domain. This domain architecture enables AhR to sense and integrate a wide array of chemical signals, thereby playing multifaceted roles in cellular metabolism, immune regulation, and tumorigenesis (Goya-Jorge et al., 2021). The ligand-binding pocket of AhR is highly hydrophobic and can accommodate structurally diverse ligands, which can be classified into four main categories: environmental pollutants (e.g., TCDD, PAHs), dietary indoles (e.g., I3C, DIM), endogenous tryptophan metabolites (e.g., kynurenine, FICZ) (Ye et al., 2022), and certain drugs. Notably, endogenous indoles and their derivatives, produced by the gut microbiota through tryptophan metabolism, play a critical role in maintaining intestinal homeostasis and influencing disease processes, including colorectal cancer (Griffith et al., 2025).

**FIGURE 1:**
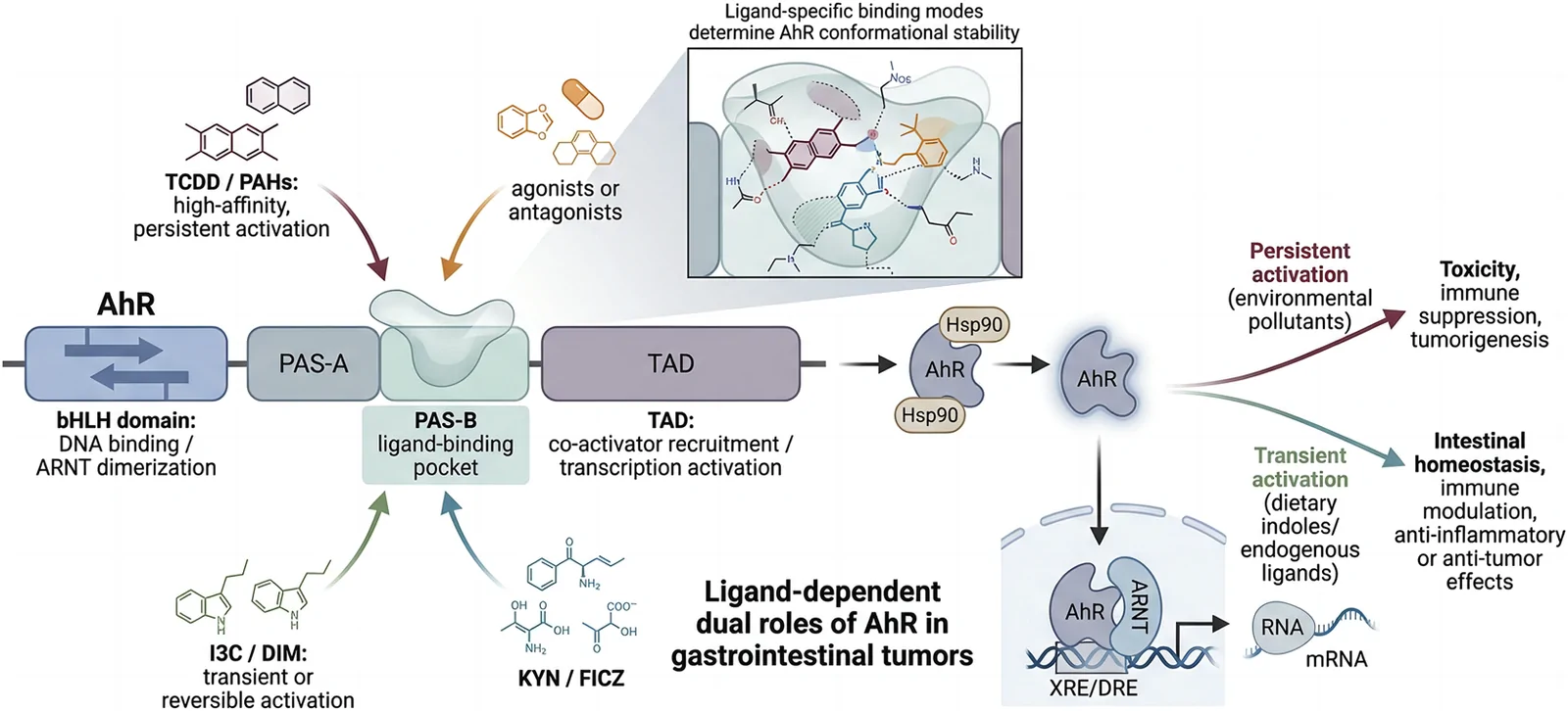
Structural basis and ligand-driven activity of AhR in gastrointestinal tumors. The AhR protein consists of bHLH, PAS-A, and PAS-B domains and a C-terminal transactivation domain. Binding of high-affinity environmental ligands (e.g., TCDD, PAHs) to the PAS-B pocket stabilizes an active conformation that drives persistent, often toxic signaling, whereas dietary and endogenous tryptophan metabolites (e.g., I3C, DIM, KYN, FICZ) typically induce transient activation. This ligand-specific signaling governs the receptor’s dual role in gastrointestinal cancers, balancing pro-tumorigenic outcomes (immune suppression, tumor progression) with homeostatic and anti-tumor effects (intestinal barrier integrity, immune modulation). Structural domain boundaries and ligand-binding data are based on experimentally validated crystal structures and biochemical studies. Pathway activation representations integrate these data into a conceptual framework. For experimental evidence by cancer type, see Table 2.

Differences in ligand binding affinity and specificity constitute the structural basis for the distinct biological effects of AhR (Zhang W. et al., 2023). Binding of environmental pollutants such as TCDD typically induces stable and persistent AhR activation, which is closely linked to pathological processes including tumorigenesis and immunosuppression. This link is further supported by clinical translational evidence: analysis of human pancreatic tissue from organ donors revealed that smokers exhibit significantly increased AhR activation compared to non-smokers, and PDAC tumors from patients with smoking history showed increased regulatory T cell accumulation (Griffith et al., 2026). AhR thus functions as an “exposome receptor” integrating environmental and endogenous signals (Coumoul et al., 2026). In contrast, certain dietary or endogenous ligands (e.g., I3C, FICZ) may trigger transient and reversible activation, and in some cases even exert antagonistic effects, potentially leading to anti-inflammatory or anti-tumor outcomes (Goya-Jorge et al., 2021). These ligand-specific variations arise from precise interactions between ligand molecules and amino acid residues within the PAS-B domain, which influence the conformational stability of the AhR-ligand complex, nuclear translocation efficiency, and the kinetics of DNA response element binding, ultimately resulting in divergent downstream gene expression profiles. The detailed mechanistic chain linking cigarette smoke-derived AhR ligands to PDAC progression through immune dysregulation is discussed in Section 3.4. To systematize the diverse ligand-AhR interactions across GI malignancies, Table 1 summarizes the major ligand categories, their binding properties, and tumor-specific functional outcomes in esophageal, gastric, hepatocellular, pancreatic, and colorectal cancers. A thorough understanding of AhR’s protein structure and ligand-binding properties is essential for elucidating its dual role in gastrointestinal cancers and for developing selective therapeutic agents (Figure 1).

**TABLE 1:** Classification of AhR ligands and their tumor-specific effects in gastrointestinal cancers.

Ligand category	Representative ligands	Binding affinity and persistence	GI cancer type	Functional effect	Key mechanism	References
Environmental Pollutants (HAHs and PAHs)	TCDD (dioxin); BaP(benzo[a]pyrene); 3-MC	Very high affinity (Kd ∼ pM-nM); metabolically stable;persistent activation	Esophageal (ESCC); Gastric; Colorectal; Pancreatic (PDAC)	Pro-tumorigenic (persistent activation)	Canonical: CYP1A1/1B1 induction bioactivates PAHs to DNA-reactive diol epoxides;Non-canonical: c-Src/EGFR/ERK1/2 drives proliferation; ESCC: Cyclin D1/c-Myc upregulation; Gastric: MMP-9 via c-Jun, invasiveness; PDAC: protumorigenic T-cell polarization	Goya-Jorge et al. (2021), Griffith et al. (2026), Huchzermeier and Van Der Vorst (2025), Chong et al. (2023), Perrot-Applanat et al. (2024), Rahmati et al. (2024)
Dietary Indoles (cruciferous vegetables)	I3C (indole-3-carbinol); DIM (3,3'-diindolylmethane); ICZ (indolo[3,2-b]carbazole)	Moderate affinity; transient activation; Rapidly metabolized; conditionally antagonistic	Colorectal; Gastric; Esophageal	Primarily anti-tumorigenic (context-dependent)	Canonical: transient AhR activation maintains barrier integrity, suppresses NLRP3 inflammasome; I3C/DIM repress ESCC proliferation, invasion, migration; I3C essential for anti-PD-1 therapy efficacy via physiological AhR activation; DIM induces G1 arrest in AGS gastric cancer cells	De Juan et al. (2025), Goya-Jorge et al. (2021), Ala (2022), Ngui et al. (2020), Elson and Kolluri (2023), Poźniak et al. (2025)
Endogenous Tryptophan Metabolites (IDO1/TDO2 pathway)	Kynurenine (Kyn); Kynurenic acid; FICZ (6-formylindolo[3,2-b]carbazole)	Kyn: low affinity, high abundance; FICZ: highest affinity (Kd ∼70 pM), transient (rapidly metabolized by CYP1A1)	Colorectal; Gastric; Hepatocellular (HCC); PDAC	Kyn: pro-tumorigenic (immunosuppression); FICZ: dose-dependent dual role	Kyn-AhR: expands Tregs, upregulates PD-L1, drives CRC liver metastasis via TDO2 ; Kyn-AhR: activates PI3K/ERK/Wnt signaling network ; HCC: TDO2 autocrine IL-6 promotes progression ; FICZ: low dose pro-inflammatory/anti-tumor; high dose immunosuppressive (like TCDD)	Wu et al. (2021), Ye et al. (2022), Ala (2021), Miyazaki et al. (2022)
Microbiota-Derived Indoles (gut bacteria)	Indole-3-acetic acid (IAA); Indole-3-propionic acid (IPA); Indole-3-lactic acid (ILA); Tryptamine	Low-moderate affinity; transient activation; tissue-specific production	Colorectal; HCC; PDAC	Predominantly anti-tumorigenic (homeostatic); context-dependent	IAA: *Lactobacillus* paracasei activates AhR/MTDH/NF-kB axis to inhibit GI tumors; IPA: *Fusobacterium* nucleatum activates macrophage AhR, drives M2 polarization; ILA: Bifidobacterium breve directs macrophage differentiation, ameliorates colitis-associated tumorigenesis; PDAC: tryptophan-derived microbial metabolites activate AhR in TAMs to suppress anti-tumor immunity	Griffith et al. (2025), Hezaveh et al. (2022), Li Y. et al. (2024), Song et al. (2025), Garcia-Villatoro et al. (2020)
Pharmacological /Synthetic Ligands	Omeprazole (PPI); Flavonoids (quercetin, kaempferol); ITE (endogenous, 2-(1'H-indole-3'-carbonyl)-thiazole-4-carboxylic acid methyl ester)	Variable affinity; SAhRM-like profile; selective modulation	ESCC; Gastric; Colorectal; HCC	Context-dependent (agonist/antagonist)	Omeprazole: AhR agonist, inhibits ESCC proliferation via cell cycle arrest; Flavonoids: exhibit agonist or antagonist activity depending on structure; Kaempferol: modulates intestinal health and AhR signaling; ITE: reduces proliferation and migration in ER^+^cancers (model for tissue-selective AhR modulation)	Wei et al. (2022), Goya-Jorge et al. (2021), Wei et al. (2025), Bai et al. (2021), Chen J. et al. (2023a)

Ligands are categorized by source (environmental, dietary, endogenous, microbiota-derived, pharmacological) with representative compounds, binding characteristics, and functional outcomes across five GI, cancer types. The context-dependent duality of AhR is reflected in the opposing effects of persistent vs. transient ligands.

### 2.2 Canonical and non-canonical signaling pathways

The canonical signaling pathway represents the core mechanism underlying AhR’s biological functions. In the absence of a ligand, AhR resides in the cytoplasm as part of a multiprotein complex with chaperones including Hsp90, XAP2, and p23, which maintain its stable conformation (Ue et al., 2020). Upon ligand binding, AhR undergoes a conformational change and translocates to the nucleus, where it heterodimerizes with the aryl hydrocarbon receptor nuclear translocator (ARNT) and binds to xenobiotic response elements (XREs) in the promoter regions of target genes (Fang et al., 2023; Wei et al., 2025). This binding initiates transcription of downstream genes, with the most classic and well-characterized target being cytochrome P450 1A1 (CYP1A1). In addition, the expression of other phase I metabolic enzymes such as CYP1B1 is directly regulated by this pathway (Park et al., 2020). Induction of these enzymes is a key component of AhR-mediated xenobiotic metabolism and detoxification, enhancing substrate water solubility through catalytic oxidation and thereby facilitating excretion (Goya-Jorge et al., 2020). Consequently, the canonical AhR/ARNT signaling pathway has long been recognized as a critical element of the adaptive defense system against environmental toxins (Kou and Dai, 2021).

Activation of the canonical AhR pathway is intimately linked to the balance between metabolic detoxification and procarcinogen activation, thereby directly influencing the initiation risk of gastrointestinal tumors. The gastrointestinal tract serves as the primary portal of exposure to exogenous substances, and AhR acts as an environmental sensor that determines the fate of procarcinogens (Huchzermeier and Van Der Vorst, 2025). On one hand, AhR activation induces enzymes such as CYP1A1 to facilitate procarcinogen detoxification, exerting a chemopreventive effect. For instance, some studies suggest that AhR may possess tumor-suppressive activity in the colon (Safe et al., 2023). On the other hand, certain procarcinogens (e.g., PAHs) themselves act as AhR ligands; their activation leads to the generation of electrophilic intermediates by phase I enzymes. If these intermediates are not promptly conjugated and detoxified, they can induce DNA damage and mutations, thereby initiating tumorigenesis (Chong et al., 2023). Moreover, the gastrointestinal tract harbors abundant endogenous ligands, primarily derived from dietary tryptophan metabolites and indole compounds produced by the gut microbiota (e.g., I3C, kynurenic acid). These ligands help maintain intestinal epithelial barrier function, immune homeostasis, and anti-inflammatory effects through AhR (Coumoul et al., 2026; Ala, 2022; Ngui et al., 2020). However, under pathological conditions such as chronic inflammatory bowel disease (IBD) or specific dysbiosis, the endogenous ligand profile is altered, leading to dysregulated AhR signaling, disruption of homeostasis, and the establishment of a pro-tumorigenic microenvironment. Thus, through precise regulation of xenobiotic metabolism and internal balance, the canonical AhR pathway plays a complex and critical role in the initiation of gastrointestinal tumors.

AhR functions extend well beyond the canonical pathway; it can also rapidly regulate cellular processes through non-canonical mechanisms. Studies have shown that AhR directly interacts with proteins such as SRC, EGFR, and NF-κB, thereby swiftly activating the corresponding signaling pathways. For example, the AhR-interacting protein AIP, a co-chaperone of AhR, participates in regulating IKK/NF-κB signaling in T cells (Kazzaz et al., 2024). Such direct interactions enable AhR to respond rapidly to environmental signals and play roles in stress responses, proliferation, and immune regulation. Furthermore, the activation status of AhR is associated with physicochemical properties such as cell membrane permeability, which can further influence the efficiency of its non-canonical pathways (Ruano et al., 2026). These non-canonical mechanisms may directly affect cancer cell invasion, metastasis, and immune evasion within the gastrointestinal tumor microenvironment.

**FIGURE 2:**
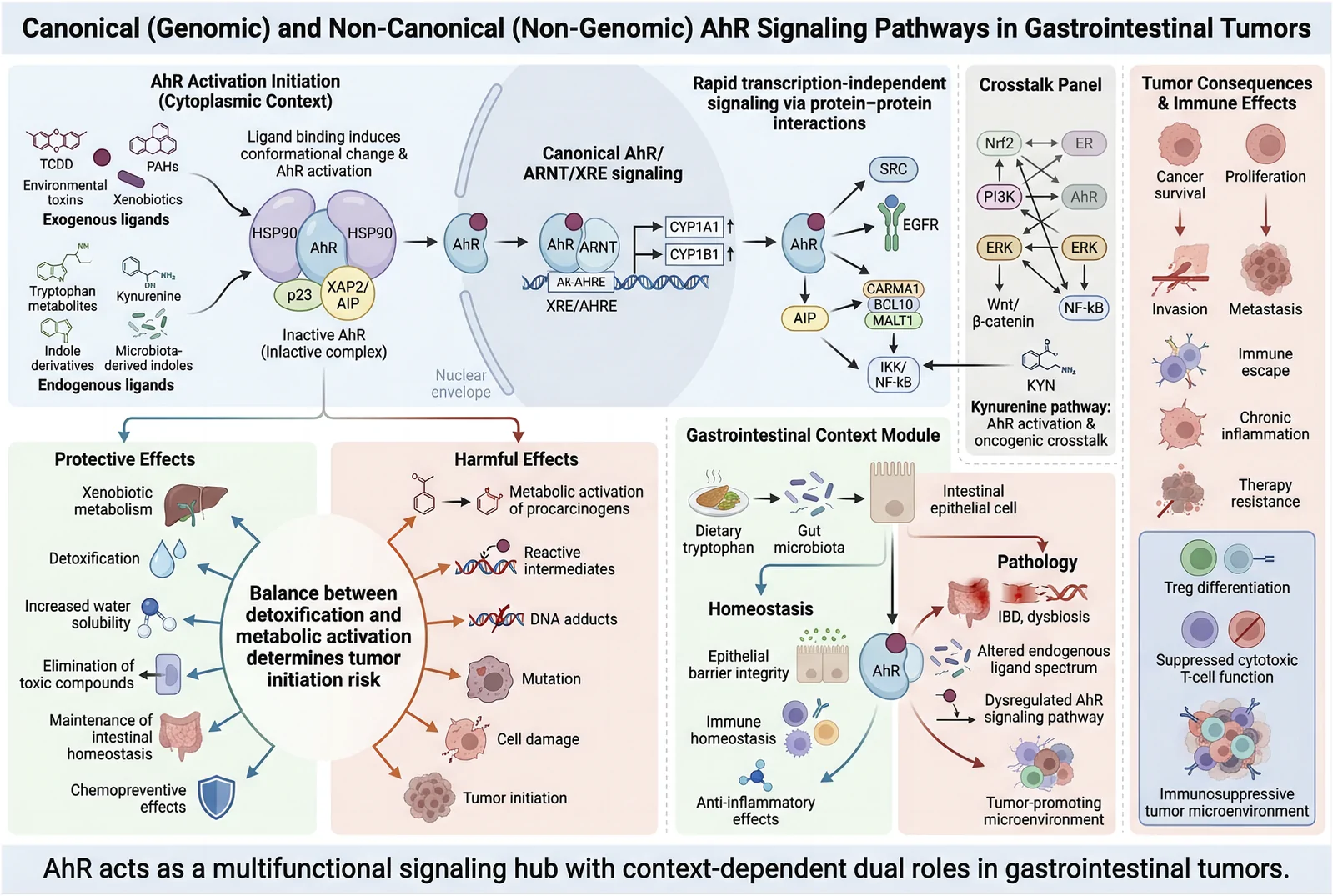
Canonical and non-canonical AhR signaling pathways in gastrointestinal cancers. **(A)** In the resting state, cytoplasmic AhR is held in an inactive complex with Hsp90, p23, and XAP2. Upon ligand binding, AhR translocates to the nucleus, heterodimerizes with ARNT, and binds xenobiotic response elements (XREs) to activate phase I/II metabolic enzymes and drug transporters. This pathway is dual-functional: it detoxifies carcinogens but can also bioactivate procarcinogens (e.g., PAHs to DNA-reactive metabolites). Negative feedback occurs via AhRR and proteasomal degradation. **(B)** Non-canonical signaling proceeds through transcription-independent mechanisms: ligand-bound AhR releases c-Src, activating EGFR–ERK1/2 signaling; AhR also heterodimerizes with NF-κB, KLF6, and pRb to regulate inflammation and cell-cycle genes, and functions as an E3 ligase targeting estrogen receptor. **(C)** Pathway crosstalk with Nrf2, estrogen signaling, and Wnt/β-catenin amplifies oncogenic signaling; kynurenine-activated AhR drives PI3K/ERK and Wnt networks. **(D)** Ligand identity determines output: dietary indoles and microbiota-derived metabolites activate homeostatic programs (barrier integrity, anti-inflammatory responses), whereas persistent pollutants and tumor-derived kynurenine drive immunosuppression and chemoresistance. The canonical AhR/ARNT/XRE pathway is well-established experimentally; non-canonical interactions are supported by *in vitro* and *in vivo* studies cited in Sections 2.2, 4.

The complexity of AhR function is further reflected in its extensive cross-talk with other transcription factors, including Nrf2 (involved in oxidative stress) and the estrogen receptor (ER). Environmental pollutants can activate AhR, inducing expression of CYP1 family enzymes and promoting the conversion of estrogens into genotoxic metabolites (Fauteux et al., 2023). In gastrointestinal tumors, the tryptophan metabolite kynurenine acts as an endogenous AhR ligand; its overactive metabolic pathway is associated with poor prognosis and closely interacts with signaling pathways such as PI3K, ERK, and Wnt/β-catenin to form a tumor-driving network (Ala, 2021). The highly context-dependent effects of AhR—varying with tissue type, ligand, and co-existing signals—are determined by this complex interplay of canonical and non-canonical pathways and extensive cross-talk. An in-depth understanding of these non-canonical mechanisms is essential for targeted therapy (Figure 2).

## 3 Expression and function of AhR in gastrointestinal tumors

AhR expression is broadly altered across gastrointestinal malignancies, yet its prognostic and functional significance varies markedly by cancer type and cellular context. Pan-cancer analysis of TCGA and GEO datasets reveals that AHR mRNA is significantly upregulated in thyroid, colon, pancreatic, and stomach tumors compared with corresponding normal tissues (Mo et al., 2021), confirming that transcriptional upregulation is a common feature of AhR in GI oncology. However, this upregulation does not uniformly predict aggressive behavior. In stomach adenocarcinoma, AHR mRNA achieves an AUC of 0.956 in distinguishing tumor from normal tissue, and high AHR expression is associated with worse overall survival (HR = 1.46) (Wan et al., 2025). Yet in colorectal cancer, despite AHR mRNA upregulation in COAD, expression level alone is not prognostic—consistent with the receptor’s context-dependent function, where identical expression levels can produce diametrically opposite outcomes depending on ligand identity and cellular context. Immunohistochemical studies further reveal cancer-specific expression patterns: in ESCC, the tumor-to-nontumor protein expression ratio for AhR is 2.2:1 (Zhu et al., 2020); AhR is highly expressed in a subset of gastric cancers and shows altered subcellular localization, supporting its potential association with oncogenic signaling (Perrot-Applanat et al., 2024); in HCC, tissue microarray analysis (n = 94) demonstrates significantly stronger AhR staining in tumor tissues compared with adjacent non-tumorous tissues (P = 0.003) and normal hepatic tissues (P = 0.004), with upregulation correlating with T stage (P = 0.03) (Liu et al., 2013), and high AHR expression predicting shorter survival in a cohort of 289 patients (Wang et al., 2017); in PDAC, elevated AhR levels correlate with worse clinical outcomes (Mei et al., 2026), and high AHR expression in tumor-associated macrophages is linked to rapid disease progression and mortality (Hezaveh et al., 2022). A critical implication of these findings is that AhR expression level alone is insufficient for patient stratification; functional context—including ligand milieu, disease stage, and microenvironmental composition—must be considered alongside expression data. The cancer-specific functional roles are detailed in the following subsections (Table 2).

**TABLE 2:** AhR functions across GI cancers.

Cancer type	AhR expression	Dominant mode	Key experimental evidence	Key ligands/Pathways	Therapeutic interventions
ESCC	Overexpressed; IHC ratio 2.2:1 (Zhu et al., 2020); associated with poor prognosis	Promoter (drives proliferation, EMT, immune evasion)	AhR knockdown inhibits proliferation (Zhu et al., 2020); DIM suppresses COX2/PGE2/STAT3 *in vivo* (Zhu et al., 2020); omeprazole inhibits ESCC via AhR (Bai et al., 2021)	AhR-EGFR crosstalk (Arciga et al., 2025); COX2/PGE2/STAT3 (Zhu et al., 2020); cyclin D1/c-Myc (Rahmati et al., 2024)	DIM (*in vivo* tumor suppression) (Zhu et al., 2020); omeprazole (*in vitro*) (Bai et al., 2021)
Gastric	AHR mRNA is elevated in TCGA-STAD and distinguishes tumor from normal tissue with an AUC of 0.956; higher expression is associated with adverse survival outcomes (Wan et al., 2025)	Gatekeeper/Promoter dynamic switch (dependent on molecular subtype)	EBV-LMP2A suppresses AhR via ERK (Jiang et al., 2021); Hp-associated AhR responds to inflammatory ligands (Pirzadeh et al., 2022); AhR knockdown reduces gastric cancer invasiveness (Perrot-Applanat et al., 2024)	Hp-AhR (Pirzadeh et al., 2022); kynurenine-AhR (Pirzadeh et al., 2022); AhR/CYP1A1	Taxifolin via AhR/CYP1A1 (*in vitro* + *in vivo*) (Xie et al., 2021); resveratrol reverses TCDD-induced CYP1A1 (Perrot-Applanat et al., 2024)
CRC	AHR mRNA upregulated (TCGA-COAD) but expression level alone not prognostic (Mo et al., 2021)	Gatekeeper → Promoter transition (dependent on disease stage and ligand)	IE-AhR-KO accelerates AOM/DSS tumorigenesis (Garcia-Villatoro et al., 2020); AhR suppresses FOXM1 (Han et al., 2020; Yang et al., 2022); kynurenine drives immune evasion (Miyazaki et al., 2022)	IDO1/TDO2 → kynurenine (Anderson, 2022); I3C/DIM → gatekeeper (Ran et al., 2026)	Compound 7k (CRC organoids) (Choi et al., 2025); CH-223191 (Li Y. et al. (2024); Kurata et al., 2023; Ichisaka et al., 2025); I3C/DIM (preclinical)
HCC	AhR protein elevated vs. peritumor (P = 0.003) and normal (P = 0.004); correlates with T stage (P = 0.03) (Liu et al., 2013)	Promoter (drives metabolic reprogramming)	AhR drives glycolipid metabolic reprogramming (Xiao et al., 2025); ZNF165 enhances CYP1A1 nuclear translocation (Liu et al., 2022)	Kynurenine-AhR (Wu et al., 2021); HBx-AhR-NF-kB (Celik-Turgut et al., 2023)	Sorafenib (AhR antagonist activity) (Wei et al., 2022); TDO inhibitor + irinotecan (Liu et al., 2021)
PDAC	AhR elevated; correlates with worse prognosis (Mei et al., 2026); protein in cytoplasm and nucleus (Griffith et al., 2026)	Promoter (bridges tumor-stroma, establishes immunosuppressive network)	AhR inhibits antigen presentation/MHC-I (Mei et al., 2026); cigarette AhR ligands→ Th22/Treg (Griffith et al., 2026); AhR→ ELAVL1 → DCK downregulation (Stukas et al., 2023)	Cigarette PAHs-AhR (Griffith et al., 2026); microbial tryptophan metabolites (Hezaveh et al., 2022)	BAY + gemcitabine (*in vitro* synergy) (Stukas et al., 2025); IK-175 (Phase I) (Aggen et al., 2026)

### 3.1 Expression and role of AhR in esophageal and gastric cancer

In esophageal squamous cell carcinoma (ESCC), AhR is frequently overexpressed, and high AhR expression is significantly associated with poor patient prognosis (Zhu et al., 2020). The underlying pro-oncogenic mechanisms include upregulation of Cyclin D1 and c-Myc, which drives G1/S phase transition and promotes proliferation (Rahmati et al., 2024), as well as enhancement of tumor cell invasion and migration through AhR-EGFR signaling cross-talk (Arciga et al., 2025). Collectively, these findings position AhR as a key “promoter” in ESCC—consistently driving proliferation, invasion, and poor prognosis through Cyclin D1/c-Myc and EGFR cross-talk—and suggest that it may represent a valuable target for prognostic prediction and therapeutic intervention.

In gastric cancer, AhR oscillates between “gatekeeper” and “promoter” modes depending on molecular subtype and environmental context. In its “promoter” capacity, high AhR expression in gastric cancer has been associated with activation of oncogenic signaling pathways (Perrot-Applanat et al., 2024), and in H. pylori-associated gastric cancer, AhR responds to ligands within the inflammatory microenvironment and synergistically promotes carcinogenesis (Pirzadeh et al., 2022). Conversely, in its “gatekeeper” capacity, AhR activation has been associated with cell-cycle arrest, apoptosis, or senescence in selected experimental contexts, potentially involving p53-related regulators such as p21 and Bax (Elson and Kolluri, 2023). The determinants of this mode-switching—ligand identity, microenvironmental cues, and cross-talk with signaling pathways such as the p53 axis—remain incompletely defined and constitute a priority for future investigation. Although esophageal and gastric cancers have less extensive AhR-targeted therapeutic data than CRC, emerging preclinical evidence—including DIM-mediated tumor suppression in ESCC (Zhu et al., 2020), taxifolin-induced AhR/CYP1A1 pathway inhibition in gastric cancer (Xie et al., 2021), and omeprazole-mediated AhR modulation in ESCC (Bai et al., 2021)—supports the biological rationale for AhR intervention in these malignancies (Table 2).

### 3.2 The context-dependent transition of AhR from “gatekeeper” to “promoter” in colorectal cancer (CRC)

In intestinal homeostasis, AhR operates in its “gatekeeper” mode—integrating environmental, dietary, and microbial signals to maintain epithelial barrier integrity, regulate intestinal stem cell function, and suppress inflammation-associated tumorigenesis (Ran et al., 2026). This gatekeeper function is contingent on transient activation by physiological ligands, including dietary I3C derived from cruciferous vegetables and indole metabolites generated by the gut microbiota from tryptophan (De Juan et al., 2025). Upon activation by these ligands, AhR is essential for maintaining intestinal epithelial barrier function, regulating immune cells, and suppressing intestinal inflammation, thereby exerting anti-tumor effects. AhR controls intestinal stem cell maintenance and differentiation, providing protection against colonic tumorigenesis (Zhang et al., 2025); conversely, AhR deficiency exacerbates colitis and associated carcinogenesis. Dietary and microbial AhR ligands are considered promising chemopreventive strategies for colorectal cancer (Ye et al., 2022; Poźniak et al., 2025), and natural compounds such as kaempferol also improve intestinal health by modulating AhR (Chen J. et al., 2023).

These findings have begun to translate into microbiome-targeted therapeutic strategies. Bifidobacterium breve-derived indole-3-lactic acid (ILA) activates AhR in macrophages to suppress colitis-associated tumorigenesis (Li Y. et al., 2024), while *Lactobacillus* gallinarum-derived ILA inhibits the IDO1/kynurenine/AhR axis in regulatory T cells, thereby enhancing anti-PD-1 efficacy in colorectal cancer models (Fong et al., 2023). Notably, *Lactobacillus* reuteri translocates to tumors and releases indole-3-aldehyde (I3A), which activates AhR in CD8^+^ T cells to promote IFN-γ production and facilitate immune checkpoint inhibitor response; a tryptophan-enriched diet potentiated these effects in preclinical melanoma models (Bender et al., 2023). These observations collectively support the concept that probiotic supplementation and dietary tryptophan enrichment may serve as adjunctive strategies to modulate AhR activity in the tumor microenvironment, although their efficacy in gastrointestinal cancers specifically requires clinical validation.

The transition from “gatekeeper” to “promoter” is not stochastic but governed by convergent shifts across several contextual dimensions. (i) Disease stage: during early, inflammation-driven carcinogenesis, dietary and microbial indole ligands dominate, maintaining tumor-suppressive output—including suppression of Wnt/β-catenin signaling in APC/KRAS-mutant stem cells (Han et al., 2021), inhibition of FOXM1-driven stemness networks (Han et al., 2020; Yang et al., 2022), and enhancement of IL-22/STAT3-mediated DNA damage repair via SOCS3 repression (Han et al., 2022). As established tumors upregulate IDO1 and TDO2, the ligand landscape shifts to persistent kynurenine-driven signaling, reprogramming AhR toward promoter output (Anderson, 2022). (ii) Ligand identity: physiological indole ligands (I3C, DIM, ILA, I3A) activate AhR transiently within a homeostatic window (De Juan et al., 2025; Li Y. et al., 2024; Wang et al., 2015; Cigliano et al., 2024), whereas tumor-derived kynurenine generates sustained activation that promotes immune evasion through a Treg-macrophage suppressive axis—reversible by selective AhR blockade and potentiated by PD-1 inhibition (Campesato et al., 2020). (iii) Cellular context: in intestinal epithelial cells and ILC3s, AhR activation reinforces barrier integrity and anti-tumor immunity (Ye et al., 2022; Poźniak et al., 2025); in tumor cells, TAMs, and Tregs, the same receptor is co-opted for PD-L1 upregulation, cancer stem cell maintenance, and immunosuppression (Anderson, 2020; Choi et al., 2025). (iv) Genetic background: APC/KRAS/p53 mutations sensitize colonic epithelium to AhR loss (Han et al., 2021; Han et al., 2020), while simultaneously creating a permissive context in which kynurenine-driven AhR activation sustains Wnt signaling and stemness. (v) Microbiome composition: protective commensals (Akkermansia muciniphila, Bifidobacterium breve, *Lactobacillus* reuteri) supply indole ligands that reinforce gatekeeper signaling (Li Y. et al., 2024; Cigliano et al., 2024; Zhang L. et al., 2023; Song et al., 2025), whereas pathogenic species (*Fusobacterium* nucleatum, *Peptostreptococcus* anaerobius) generate tumor-promoting metabolites that shift AhR toward promoter activity (Ternes et al., 2022).

### 3.3 Hepatocellular carcinoma and AhR-driven metabolic reprogramming

In hepatocellular carcinoma (HCC), AhR predominantly operates in its “promoter” mode, driving metabolic reprogramming that sustains tumor cell survival and proliferation. HCC is frequently accompanied by profound metabolic remodeling, and high expression or activation of AhR is a key driving force (Xiao et al., 2025). Gut microbiota dysbiosis impairs tryptophan metabolism and activates AhR, thereby promoting liver tumorigenesis (Chen et al., 2022). Dysregulation of the tryptophan/kynurenine/AhR/CYP1A1 axis accelerates HCC cell proliferation and migration (Liu et al., 2022). AhR directly regulates the transcription of key glycolytic enzymes, including HK2 and LDHA, thereby enhancing the Warburg effect, and upregulates SLC1A5 and GLS to promote glutaminolysis, supporting tumor cell survival under nutrient-deficient conditions. Additionally, AhR influences xenobiotic metabolism through regulation of enzymes such as CYP1A1, as demonstrated in HepG2 cells (La et al., 2025); notably, halogenated indole agonists persistently induce CYP1A1 activity (Vrzalová et al., 2024). Glycogen synthase kinase 3β (GSK3β)-mediated phosphorylation can target AhR for lysosomal degradation, acting as an “off” switch for its activity—a mechanism also observed in Hep3B cells (Yang and Chan, 2021). Collectively, these findings indicate that AhR drives a pro-tumorigenic metabolic environment from the early stages of HCC.

AhR also indirectly promotes HCC progression by shaping the tumor microenvironment. Aberrant AhR activation can induce hepatic stellate cell activation, thereby promoting liver fibrosis and the formation of a pro-tumorigenic microenvironment. The transcription factor ZNF165 enhances AhR activity by facilitating CYP1A1 nuclear translocation (Liu et al., 2022). Cross-talk between AhR and HBx or NF-κB is particularly critical in HBV-associated HCC (Celik-Turgut et al., 2023). Notably, the phytochemical p-hydroxycinnamic acid (HCA) can transiently activate AhR in a “gatekeeper”-like manner to inhibit HepG2 cell growth (Yamaguchi et al., 2021), illustrating that even in HCC—where AhR is predominantly a “promoter”—context-specific ligand choice can partially restore gatekeeper function, albeit without achieving the sustained homeostatic signaling seen in normal intestinal epithelium. AhR inhibitors or modulators, such as the FOXM1 inhibitor FDI-6, can also activate AhR and suppress tumor spheroid formation (Yamashita et al., 2023), offering new therapeutic avenues for HCC. The preclinical evidence for AhR-driven metabolic reprogramming in HCC is substantiated by the AhR antagonist activity of the approved multi-kinase inhibitor sorafenib (Wei et al., 2022) (Table 2).

However, the mechanistic understanding of AhR-driven metabolic reprogramming in HCC remains constrained by the limitations of current preclinical models. A substantial proportion of the evidence—including the TDO2/kynurenine/AhR axis, the IDO1/AhR/β-catenin crosstalk, and the ZNF165-mediated AhR enhancement—was derived from immortalized cell lines (primarily HepG2 and SMC-7721) and subcutaneous xenograft models. Microarray profiling has revealed that HepG2 cells and their xenograft counterparts are vastly different from primary HCC tumors at the transcriptomic level, and even patient-derived xenografts (PDXs) diverge significantly from their originating tumors (Wang et al., 2015). Only approximately 45% of agents showing xenograft responses ultimately exhibit clinical activity, and the sensitivity of hepatoma cell lines to sorafenib in preclinical assays far exceeds the clinical response rate of 43% (Cigliano et al., 2024). Furthermore, subcutaneous xenograft models lack the liver-specific microenvironmental components—hepatic stellate cells, Kupffer cells, and liver sinusoidal endothelial cells—that are integral to AhR-mediated TME remodeling. Establishing causal relationships between AhR activation and specific metabolic programs will require validation in more physiologically faithful systems, including patient-derived organoids (PDOs) with stromal co-culture and orthotopic models that preserve the hepatic niche.

### 3.4 Specificity of AhR–stroma interactions in pancreatic ductal adenocarcinoma

Pancreatic ductal adenocarcinoma (PDAC) is characterized by a highly fibrotic and immunosuppressive tumor microenvironment (TME), which contributes to treatment resistance and poor prognosis (Li X. et al., 2024). Within this TME, AhR is markedly activated and functions exclusively in its “promoter” mode—serving as a critical bridge that connects tumor cells with stromal cells to systematically establish an immunosuppressive network. In PDAC cells, AhR epigenetically suppresses MHC-I expression, thereby impairing antigen presentation and facilitating immune evasion (Mei et al., 2026); concurrently, AhR disrupts the nucleocytoplasmic shuttling of ELAVL1, reducing sensitivity to gemcitabine (Stukas et al., 2023). In cancer-associated fibroblasts (CAFs), AhR activation promotes the secretion of IL-6 and TGF-β, driving fibrosis and suppressing T-cell function (Hezaveh et al., 2022). Moreover, indole-derived compounds produced by the gut microbiota from tryptophan can activate AhR in tumor-associated macrophages (TAMs), inhibiting CD8^+^ T cells. AhR ligands derived from cigarette smoke polarize CD4^+^ T cells toward TH22 and regulatory T (Treg) subsets, weakening CD8^+^ T cell effector function and promoting PDAC progression (Griffith et al., 2026)—consistent with the smoking/AhR activation observation noted in Section 2.1. Thus, AhR systematically establishes an immunosuppressive network by acting on tumor cells, CAFs, TAMs, and T cells, leading to poor responses to immunotherapy in PDAC (Chouari et al., 2023). Notably, in contrast to CRC—where AhR transitions from “gatekeeper” to “promoter” during disease progression—in PDAC, AhR appears to lack a significant gatekeeper phase, possibly because the pancreas is not a primary mucosal barrier tissue and lacks the dietary/microbial AhR ligand repertoire that sustains intestinal homeostasis. The therapeutic armamentarium for AhR targeting in PDAC is currently limited to preclinical synergy data [BAY + gemcitabine (Stukas et al., 2025)] and early-phase clinical results [IK-175 (Mcgovern et al., 2022)]; nevertheless, the stroma-dependent promoter function of AhR in PDAC provides a distinct mechanistic rationale that differs from CRC’s gatekeeper-to-promoter transition (Table 2).

The mechanistic insights into AhR-stroma interactions in PDAC must be interpreted in light of significant preclinical model limitations. The AhR-mediated MHC-I suppression, ELAVL1/DCK regulation, and TAM-specific AhR activation have been characterized primarily using 2D cell lines and xenograft models, which fail to recapitulate the defining features of human PDAC: the dense desmoplastic stroma, the complex CAF heterogeneity, and the immunosuppressive TME (Suri et al., 2020). Xenograft models necessitate immunocompromised hosts, precluding assessment of AhR-dependent immune cell crosstalk that is central to PDAC progression. Although genetically engineered mouse models (GEMMs), such as the KPC model, preserve immune competence, the desmoplastic stroma in these mice never reaches the extreme levels observed in human tumors, likely because the tumorigenic process in mice unfolds over weeks rather than the decades required in humans. Moreover, GEMMs introduce multiple genetic insults simultaneously, whereas human PDAC develops through stepwise accumulation of KRAS, TP53, CDKN2A, and SMAD4 alterations. PDOs retain tumor cell heterogeneity but progressively lose stromal and immune components during culture, limiting their utility for studying AhR-TME interactions. Consequently, the AhR-centered immunosuppressive network proposed here awaits validation in multi-component co-culture systems incorporating CAFs, immune cells, and organoids within biomimetic matrices, as well as in humanized mouse models that more faithfully recapitulate the PDAC microenvironment.

## 4 Core molecular mechanisms of AhR in regulating gastrointestinal tumor progression

### 4.1 Regulation of tumor cell stemness and epithelial–mesenchymal transition

AhR regulates tumor cell stemness and epithelial–mesenchymal transition (EMT) during gastrointestinal tumor development. In colorectal cancer (CRC), AhR acts as a tumor suppressor, and its deletion promotes tumorigenesis. Intestinal epithelial-specific knockout of AhR enhances the expansion of colonic stem/progenitor cells carrying Apc/Kras mutations, a mechanism involving upregulation of Wnt signaling. In the Apc^S580/+^; Kras^G12D/+^ mouse model, AhR deficiency promotes cecal and colonic tumorigenesis by enhancing Wnt signaling (Han et al., 2021). Furthermore, in a colitis-associated colorectal cancer model, AhR deficiency enhances stem cell proliferation and tumor growth, and its anti-tumor activity depends on inhibition of FOXM1 and its downstream targets (Han et al., 2020); single-cell transcriptomics revealed that AhR deficiency increases the expression of FOXM1-regulated genes across various colonic cell types (Yang et al., 2022). Collectively, these findings indicate that AhR limits tumor stemness by suppressing stemness-associated networks such as Wnt and FOXM1.

AhR also regulates EMT. *Fusobacterium* nucleatum activates macrophage AhR through its metabolite 3-indolepropionic acid, driving M2 polarization and promoting immunosuppression, thereby indirectly facilitating tumor migration and invasion (Song et al., 2025). Non-canonical AhR signaling interacts with EMT-related pathways, including TGF-β, SNAIL, and ZEB1 (Sondermann et al., 2023). In gastric cancer, *H. pylori* infection downregulates AhR and its inhibitory factors while increasing inflammatory cytokines; concurrently, the tryptophan metabolite kynurenine activates AhR, leading to immunosuppression. Collectively, these factors drive EMT and tumor progression. Thus, AhR functions as a hub that integrates microenvironmental, microbial metabolic, and inflammatory signals to regulate stemness and EMT in gastrointestinal tumor cells.

### 4.2 Remodeling of the tumor immune microenvironment

AhR plays a key role in remodeling the immune microenvironment of gastrointestinal tumors by modulating immune cell functions and influencing tumor immune evasion (Li et al., 2025). AhR activation enhances the immunosuppressive functions of myeloid-derived suppressor cells (MDSCs), regulatory T cells (Tregs), and tumor-associated macrophages (TAMs), thereby dampening anti-tumor immune responses (Campesato et al., 2020). In colorectal cancer, AhR activation not only directly suppresses effector immune cells but also shapes an immunosuppressive network that promotes tumor progression (Swain et al., 2025). The tryptophan metabolite kynurenine (KYN), as an endogenous AhR ligand, induces IDO1 expression, establishing an AhR–IDO1–KYN positive feedback loop that persistently amplifies immunosuppression. The small-molecule AhR inhibitor IK-175 blocks this pathway, reverses immunosuppression, and exhibits synergistic anti-tumor effects when combined with anti-PD-1 antibodies (Li et al., 2025). Furthermore, microbial metabolites such as formate, produced by *Fusobacterium* nucleatum, drive colorectal cancer progression and enhance cancer stem cell properties through AhR activation (Ternes et al., 2022). Collectively, these findings position AhR as a central hub linking the gut microbiota, immune microenvironment, and tumor progression, making AhR a promising target for immunotherapy of gastrointestinal tumors.

### 4.3 Impact on tumor metabolism and chemotherapy resistance

AhR regulates metabolic reprogramming and chemotherapy resistance in gastrointestinal tumors. AhR activation modulates genes involved in glycolysis, glutamine metabolism, and mitochondrial function, enabling tumor cells to adapt to nutrient-deficient microenvironments. In colorectal cancer, AhR serves as a critical link between inflammation and metabolism, facilitating tumor growth and immune evasion (Swain et al., 2025). AhR maintains mitochondrial function by regulating the mitophagy receptor BNIP3; its deficiency leads to increased reactive oxygen species (ROS) and impaired respiration (Heo et al., 2023). Overactivation of the kynurenine pathway not only continuously activates AhR but also synergistically drives metabolic reprogramming through pathways such as PI3K/AKT, ERK, and Wnt/β-catenin (Ala, 2021). The gut microbial metabolite indole-3-acrylic acid influences ferroptosis sensitivity via the AhR–ALDH1A3–FSP1/CoQ axis (Cai Y. et al., 2025). Collectively, AhR integrates environmental, dietary, and microbial signals, acting as a pivotal node in the metabolic network of gastrointestinal tumors.

**FIGURE 3:**
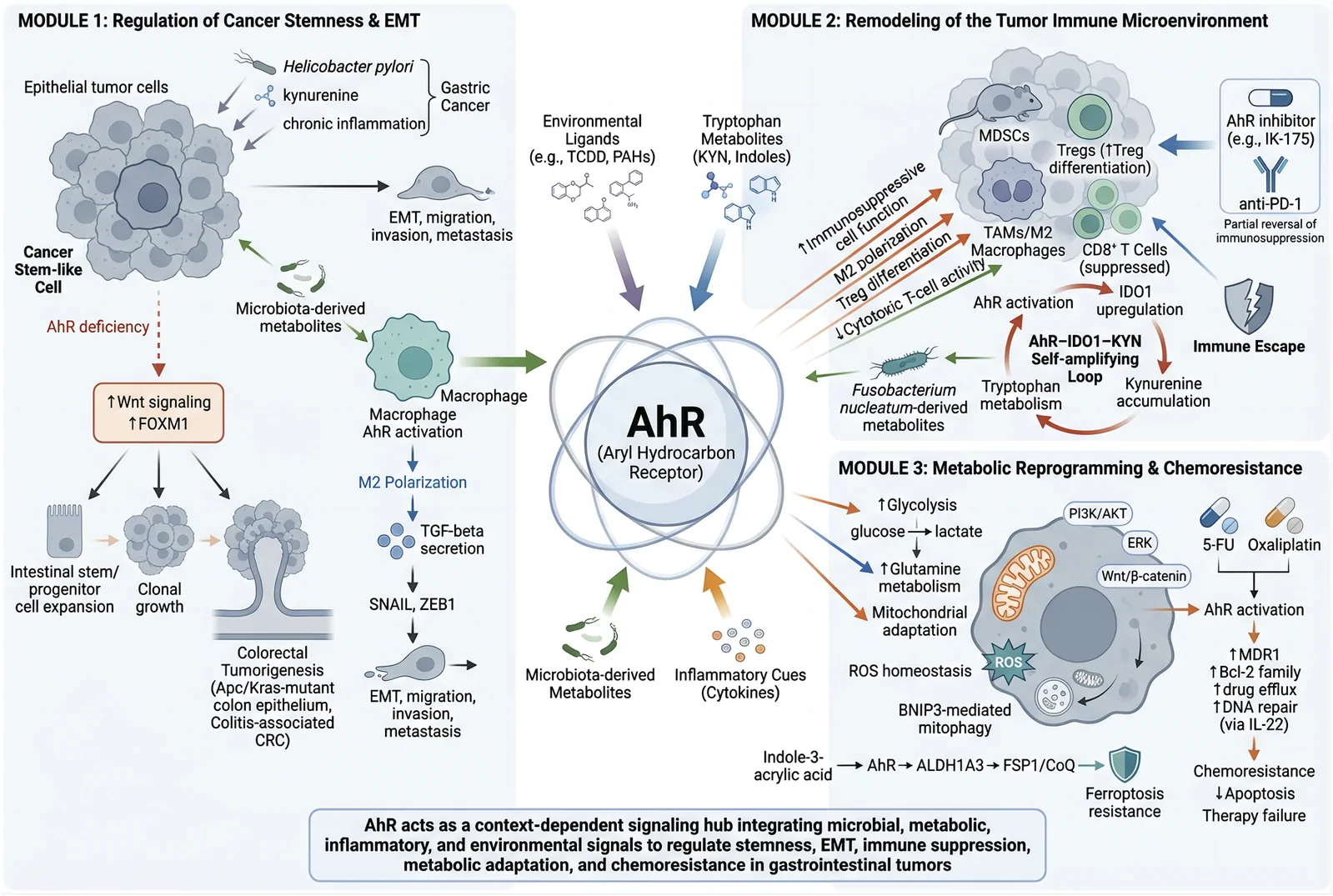
Core mechanisms of AhR-driven gastrointestinal tumor progression. AhR acts as a context-dependent signaling integrator governing three principal malignant programs. (Module 1) Regulation of cancer stemness and EMT: Loss of AhR in the intestinal epithelium potentiates Wnt/FOXM1 signaling to expand tumor-initiating cells, while microbiota-derived metabolites modulate EMT. (Module 2) Remodeling of the tumor immune microenvironment: Activated AhR expands Tregs, polarizes M2 macrophages, and suppresses CD8^+^ T cells via the IDO1–kynurenine self-amplifying loop, fostering immune escape. (Module 3) Orchestration of metabolic reprogramming and chemoresistance: AhR coordinates metabolic reprogramming (glycolysis, glutaminolysis) and chemoresistance by upregulating drug efflux proteins (MDR1) and DNA repair (via IL-22), while modulating ferroptosis sensitivity through the AhR–ALDH1A3–FSP1/CoQ axis. Pharmacological AhR inhibition (e.g., IK-175) can reverse these pro-tumorigenic processes. Each mechanistic module is supported by experimental evidence at varying levels of validation across GI cancer types, as detailed in Table 2.

AhR activation is also a key mechanism underlying chemotherapy resistance. AhR upregulates multidrug resistance proteins (e.g., MDR1) and anti-apoptotic proteins (e.g., Bcl-2), thereby enhancing drug efflux and resistance to apoptosis (Bajraktari-Sylejmani et al., 2025; Al-Dhfyan et al., 2023). It also helps tumor cells evade platinum-induced DNA damage by activating DNA repair pathways (e.g., IL-22-mediated repair) (Han et al., 2022). Moreover, various AhR ligands derived from the environment, diet, and microbiota sustain a pro-resistant tumor microenvironment (Vázquez-Gómez et al., 2023). In most gastrointestinal tumors, sustained AhR signaling promotes disease progression and drug resistance. Thus, targeting AhR (e.g., with antagonists) represents a potential strategy to overcome chemotherapy resistance (Dvořák et al., 2025) (Figure 3).

## 5 Development strategies for AhR-targeted anti-gastrointestinal tumor drugs

### 5.1 AhR antagonists/inverse agonists

The core strategy of AhR antagonists or inverse agonists is to block aberrant activation of AhR by pro-oncogenic ligands, thereby reversing its immunosuppressive and pro-oncogenic functions within the tumor microenvironment. In gastrointestinal tumors, the tryptophan metabolite kynurenine persistently activates AhR, leading to immunosuppression and immune evasion (Pirzadeh et al., 2022). The representative compound CH-223191 blocks AhR activation induced by gut microbial metabolites (e.g., skatole, indoxyl sulfate, indole-3-lactic acid), suppresses downstream TNF-α and IL-6 expression, and inhibits cancer cell proliferation (Li Y. et al., 2024; Kurata et al., 2023; Ichisaka et al., 2025). The novel highly selective antagonist IK-175 inhibits AhR activity in preclinical models, reduces immunosuppressive cytokines, and increases pro-inflammatory factors; it demonstrates anti-tumor activity both as a single agent and in combination with anti-PD-1 in colorectal cancer models (Mcgovern et al., 2022). Beyond CRC, AhR antagonism has been explored in other GI malignancies at the preclinical level. In ESCC, the acid suppressor omeprazole inhibits proliferation, migration, and invasion via AhR pathway modulation (Bai et al., 2021), and DIM suppresses tumor growth *in vivo* through COX2/PGE2/STAT3 repression (Zhu et al., 2020). In HCC, the approved multi-kinase inhibitor sorafenib has been confirmed to possess AhR antagonist activity (Wei et al., 2022), suggesting that AhR inhibition may contribute to its therapeutic mechanism (Table 3).

**TABLE 3:** AhR-targeted therapeutic evidence across GI cancers.

Drug category	Representative compound	Evidence stage	GI cancer Type(s)	Key finding	Limitations/Caveats
AhRAntagonist/Inverse Agonist	CH-223191	Preclinical	CRC	Blocks microbial metabolite AhR activation; inhibits TNF-a/IL-6 (Li Y. et al., 2024; Kurata et al., 2023; Ichisaka et al., 2025)	Only *in vitro*/mouse models
AhR Antagonist/Inverse Agonist	IK-175	Phase I/1b clinical	Multiple solid tumors + urothelial	40% DCR combined with nivolumab (Aggen et al., 2026)	No GI cancer Phase II data yet
AhR Antagonist (dual)	Sorafenib (multi-kinase + AhR antagonist)	Approved (HCC)	HCC	AhR antagonist activity confirmed (Wei et al., 2022)	Multi-target; not AhR-specific
AhR Antagonist	Compound 7k (pyrazolopyrimidine)	Preclinical	CRC	CRC organoid + zebrafish; selective AhR inhibition (Choi et al., 2025)	Preclinical only
SAhRM	DIM/I3C	Preclinical	ESCC, CRC	Suppresses COX2/PGE2/STAT3 in ESCC (Zhu et al., 2020); anti-proliferative in CRC	No GI malignancy RCT
SAhRM	Omeprazole	Preclinical	ESCC	Inhibits ESCC proliferation/migration via AhR (Bai et al., 2021)	*In vitro* only
SAhRM	HCA (p-hydroxycinnamic acid)	Preclinical	HCC	Transient AhR activation; anti-tumor (Yamaguchi et al., 2021)	*In vitro* only
SAhRM	Taxifolin	Preclinical	Gastric	AhR/CYP1A1 pathway; *in vivo* tumor suppression (Xie et al., 2021)	Preclinical
AhR PROTAC	No AhR-PROTAC in GI cancers	Conceptual	None	Forward-looking; based on broader PROTAC paradigm (Cai J. et al., 2025)	Complete lack of GI validation
Combination: AhR antagonist + gemcitabine	BAY + gemcitabine	Preclinical	PDAC	Upregulates DCK; synergistic enhancement (Stukas et al., 2025)	*In vitro* only
Combination: AhR antagonist + anti-PD-1	IK-175 + nivolumab	Phase I	Urothelial (not GI)	40% DCR (Aggen et al., 2026)	No GI-specific data; dietary AhR ligands essential for anti-PD-1 efficacy (De Juan et al., 2025)
Combination: AhR modulator + chemotherapy	AhR inhibitor + irinotecan	Preclinical	HCC	TDO inhibitor enhances irinotecan (Liu et al., 2021)	Preclinical

Early clinical results, however, have tempered expectations. In a first-in-human phase 1/1b trial (NCT04200963), IK-175 monotherapy and combination with nivolumab demonstrated favorable tolerability with no dose-limiting toxicities; target engagement was confirmed by *ex vivo* AhR activation assays and dose-dependent modulation of AhR-regulated genes in tumor biopsies. In checkpoint inhibitor-refractory urothelial carcinoma, the combination achieved a disease control rate of 40% (vs. 20% monotherapy), including one complete response and durable stable disease up to 74 weeks (Aggen et al., 2026). These results underscore a significant gap between preclinical efficacy and clinical outcomes, likely attributable to compensatory activation of alternative tryptophan-metabolizing enzymes (e.g., TDO2, IL4I1) and the absence of biomarker-based patient selection. Notably, these clinical data align with observations that increasing AhR inhibitor doses does not linearly improve anti-tumor outcomes in preclinical systems, likely because AhR signaling exerts both beneficial and detrimental effects whose balance varies by context (Polonio et al., 2025). Furthermore, the failure of the ECHO-301 Phase III trial for the IDO1 inhibitor epacadostat—an upstream node in the tryptophan–kynurenine–AhR axis—highlighted the risks of inadequate patient stratification, compensatory pathway activation via TDO2 and IL4I1, and the paradoxical finding that some IDO inhibitors themselves can activate AhR (Muller et al., 2019; Uyttenhove et al., 2003). These collective experiences underscore that AhR antagonism, while mechanistically rational, requires biomarker-guided patient selection and rational combination strategies to achieve clinically meaningful efficacy in gastrointestinal cancers.

The development of AhR antagonists faces significant challenges, the core difficulty being the design of highly selective drugs that avoid interfering with the physiological functions of AhR in normal tissues—such as maintaining the intestinal epithelial barrier, regulating metabolic enzymes, and contributing to immune homeostasis (Ran et al., 2026). Complete inhibition could lead to risks including exacerbated intestinal inflammation and metabolic disturbances. Future efforts should focus on developing “context-dependent” antagonists, combined with precise biomarker screening to identify patient populations most likely to benefit. Additionally, determining the optimal timing for combination with immune checkpoint inhibitors or chemotherapy will be essential to maximize efficacy while minimizing toxicity (Mcgovern et al., 2022; Chen F. et al., 2023).

### 5.2 Selective AhR modulators

Selective AhR modulators (SAhRMs) exploit “ligand-biased signaling” to selectively induce beneficial transcriptional programs while avoiding classical toxic effects. Traditional potent AhR ligands, such as TCDD, activate the receptor but are associated with severe hepatotoxicity, immunosuppression, and teratogenicity (Zhang W. et al., 2022; Zhang W. et al., 2023). SAhRMs are conceptually designed to fine-tune AhR conformation to selectively trigger anti-tumor transcriptional programs—including cell cycle arrest, induction of differentiation, and immune activation—while theoretically circumventing TCDD-like toxicity (Goya-Jorge et al., 2020). However, it is important to note that the selective modulation of AhR by SAhRMs has been demonstrated primarily in cell-based reporter assays and preclinical models, and the extent to which ligand-biased signaling can be reliably exploited in the heterogeneous tumor microenvironment of gastrointestinal cancers remains to be validated. The development of SAhRMs with well-defined functional selectivity profiles is still in its early stages, and no SAhRM has yet entered clinical trials for GI malignancies (Griffith and Frankel, 2024). This strategy relies on the ability of distinct ligands to recruit different sets of co-regulators. The AhR-interacting protein AIP influences AhR nuclear translocation and transcriptional activity, providing a theoretical basis for developing SAhRMs that modulate AhR–AIP interactions.

Sources of SAhRMs include natural product derivatives and synthetic compounds. Plant-derived flavonoids are important natural ligands that exhibit agonist or antagonist activities depending on their structure and cellular context. I3C from cruciferous vegetables and its dimer 3,3′-diindolylmethane (DIM) can activate AhR, demonstrating anti-proliferative and pro-apoptotic effects in preclinical models. Taxifolin suppresses gastric cancer progression via AhR/CYP1A1 modulation in both *in vitro* and *in vivo* models (Xie et al., 2021), and p-hydroxycinnamic acid (HCA) transiently activates AhR in HCC cells with anti-tumor effects (Xiao et al., 2025) (Table 3). However, these findings have not been validated in randomized clinical trials for gastrointestinal malignancies, and the bioavailability of dietary AhR ligands remains a significant translational challenge. Critically, recent preclinical evidence using dietary intervention models has shown that physiological levels of diet-derived AhR ligands, including I3C, are essential for the optimal efficacy of anti-PD-1 therapy: mice fed a diet naturally poor in AhR ligands exhibited significantly impaired checkpoint blockade response, which was restored by I3C supplementation through AhR-dependent reinvigoration of progenitor exhausted CD8 T cells (De Juan et al., 2025). Analysis of human NSCLC patient data further confirmed that AhR-dependent gene signatures positively correlate with anti-PD-1 treatment response, providing translational rationale for dietary AhR ligand supplementation as an adjunct to immunotherapy. However, despite these promising preclinical signals, no I3C or DIM formulation has yet entered clinical trials specifically for gastrointestinal cancers. Completed clinical trials of DIM in other cancer types—including a phase III trial for cervical dysplasia (NCT00212381) and phase II trials for prostate intraepithelial neoplasia—have yielded mixed results, highlighting the need for optimized formulations with improved bioavailability and for biomarker-driven patient selection in future GI cancer trials.

### 5.3 AhR degraders (PROTACs)

The dual tumor-suppressive and pro-oncogenic roles of AhR in gastrointestinal tumors pose a significant challenge for targeted therapy (Safe et al., 2023; Rejano-Gordillo et al., 2022). Traditional agonists or antagonists only modulate transcriptional activity without eliminating the AhR protein itself, potentially leading to therapeutic dilemmas. PROTAC technology has been proposed as a conceptual alternative: a PROTAC molecule would bind AhR at one end and recruit an E3 ubiquitin ligase at the other, theoretically leading to selective degradation of AhR via the ubiquitin-proteasome system. However, no AhR-targeted PROTAC has been reported in the context of gastrointestinal cancers to date. The discussion that follows is therefore forward-looking and based on the broader PROTAC paradigm rather than direct experimental evidence in GI malignancies. The feasibility of this approach is supported by the successful development of PROTACs targeting other nuclear receptors, but significant challenges—including tissue-specific delivery, potential disruption of AhR’s physiological roles in intestinal barrier maintenance and hepatic metabolism, and the complete lack of *in vivo* proof-of-concept in GI cancer models—must be addressed before this strategy can advance toward clinical evaluation.

The feasibility of nuclear receptor degradation by PROTAC technology has been validated in other oncological contexts. PROTACs targeting the androgen receptor, including ARV-110, have entered clinical development (Cai J. et al., 2025). These precedents establish that nuclear receptors—with their ligand-binding domains, defined crystal structures, and available antagonist warheads—are tractable PROTAC targets. Translating this paradigm to AhR would require: (i) selection of an appropriate warhead from known AhR ligands—agonists such as etoricoxib (Fang et al., 2023) and dexlansoprazole (Wei et al., 2025), or antagonists such as sorafenib (Wei et al., 2022), could serve as starting points; (ii) optimization of linker length and composition to enable productive ternary complex formation between AhR, the PROTAC, and the recruited E3 ligase (VHL or CRBN); and (iii) screening for degradation efficiency (DC50 and Dmax) in AhR-expressing GI cancer cell lines. A critical distinction must be noted: unlike ER or AR, which are largely dispensable in most non-target tissues, AhR plays essential physiological roles in intestinal barrier maintenance, hepatic metabolic homeostasis, and immune regulation (Ran et al., 2026; Li et al., 2025). Systemic AhR degradation would therefore risk broader toxicity than selective ER or AR degradation—potentially disrupting intestinal barrier integrity, exacerbating hepatic metabolic disturbances, and impairing immune surveillance (Griffith and Frankel, 2024). This differential risk profile necessitates tissue-targeted delivery strategies (e.g., prodrug approaches, antibody-PROTAC conjugates, or tumor-specific conditional activation) before clinical translation of AhR-PROTACs can be contemplated.

### 5.4 Combination strategies of AhR-targeted drugs with existing therapies

Combining AhR antagonists with immune checkpoint inhibitors aims to relieve AhR-mediated immunosuppression and enhance the efficacy of immunotherapy in “cold” tumors such as PDAC and HCC. Conversely, this strategy must be balanced against emerging evidence that physiological AhR activation by diet-derived ligands is itself essential for anti-PD-1 efficacy: dietary AhR ligand deprivation impairs checkpoint blockade response, while I3C supplementation restores anti-PD-1-mediated CD8 T cell reinvigoration (De Juan et al., 2025). These findings imply that systemic AhR blockade may inadvertently compromise the very immune mechanisms that anti-PD-1 therapy relies upon, reinforcing the need for context-specific dosing, temporal sequencing (e.g., pulsed rather than continuous AhR inhibition), and biomarker-guided patient stratification. In HCC, AhR participates in oxidative-stress-triggered TET2 activation in B cells, promoting the generation of IL-10^+^ regulatory B cells and thereby suppressing anti-tumor immunity (Lu et al., 2023). Inhibition of TET2 activity restores immune responses and enhances anti-PD-1 efficacy. In gastric cancer, *H. pylori* infection and kynurenine induce immunosuppression through AhR, increasing disease susceptibility (Pirzadeh et al., 2022); these findings further support the rationale for combining AhR-targeted therapy with immunotherapy.

**FIGURE 4:**
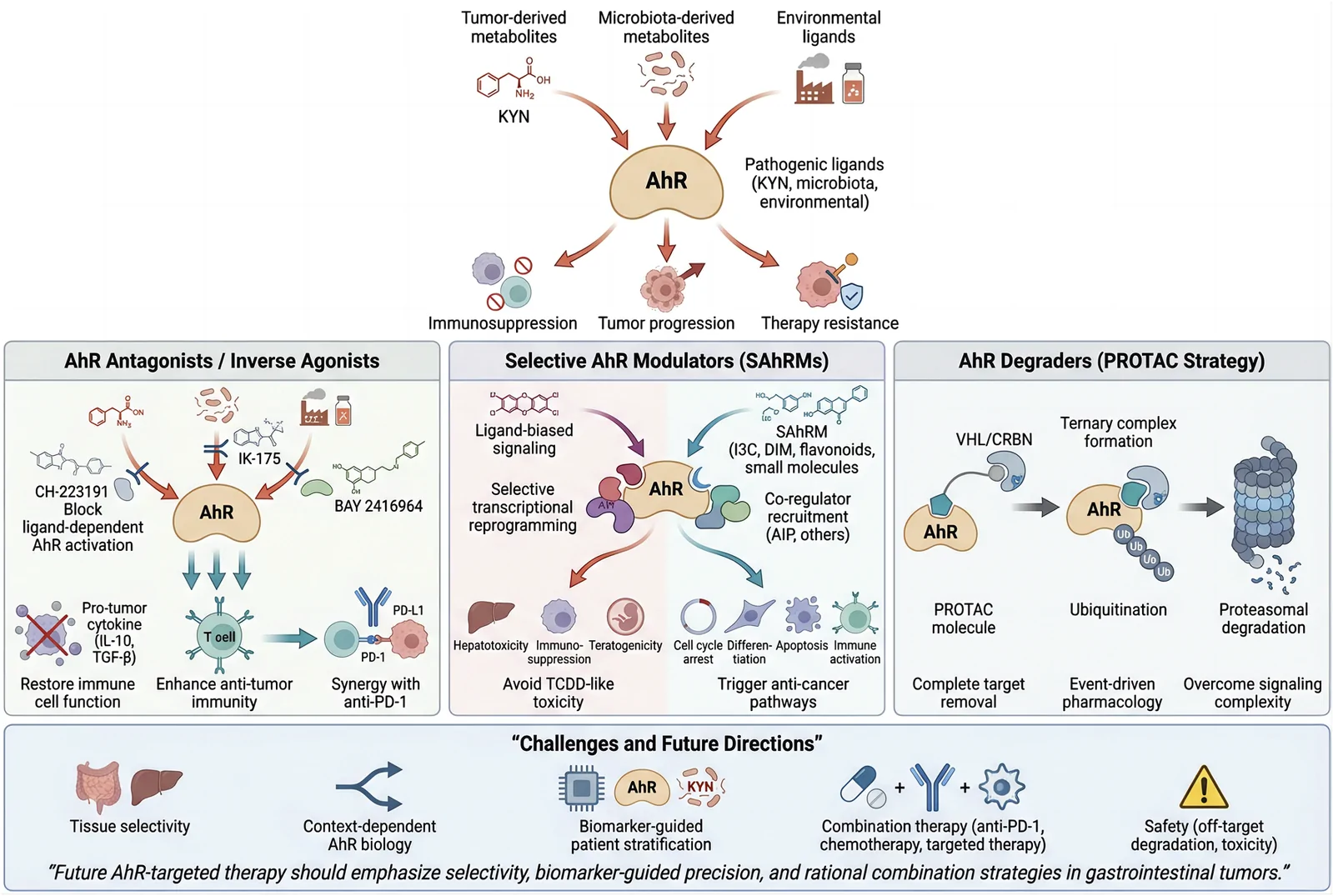
Emerging therapeutic strategies targeting AhR in gastrointestinal cancers. Strategies to target AhR in GI tumors encompass three evolving paradigms (Left) Classical antagonists/inverse agonists (e.g., CH-223191, IK-175, BAY 2416964) block the binding of pathogenic ligands (e.g., kynurenine) to reverse AhR-driven immunosuppression and enhance antitumor immunity. (Middle) Selective AhR modulators (SAhRMs) exploit ligand-biased signaling to trigger therapeutic transcriptional programs (e.g., cell cycle arrest, differentiation) while avoiding classic TCDD-like toxicities. (Right) PROTAC-based AhR degraders redirect the receptor for proteasomal degradation, enabling event-driven pharmacology that eliminates all AhR functions. Successful clinical translation requires addressing ligand promiscuity, ensuring tissue selectivity, identifying predictive biomarkers, and rational combination with anti-PD-1 therapy or chemotherapy. Antagonists and SAhRMs have preclinical and/or early clinical evidence in specific GI cancers (see Table 3). PROTAC-based strategies are conceptual/forward-looking, with no AhR-PROTAC yet reported in GI cancers. Microbiome-based interventions have preclinical evidence with emerging clinical data.

Combining AhR modulators with chemotherapy or targeted agents aims to reverse AhR-mediated drug resistance. In PDAC, AhR activity influences ELAVL1 localization and deoxycytidine kinase (DCK) expression. Inhibition of AhR synergistically enhances gemcitabine efficacy, whereas AhR activation exerts an antagonistic effect (Stukas et al., 2025). Tumor tissues exhibit a 2.2-fold increase in AhR levels and a 36% decrease in DCK expression, suggesting that AhR overactivation promotes resistance by downregulating DCK. Combination of an AhR inhibitor (e.g., BAY) with gemcitabine upregulates DCK and shows promise in overcoming resistance in preclinical models, though this has not been validated in clinical studies. In colorectal cancer, skatole promotes proliferation through both AhR-dependent and independent pathways; indole-3-acetic acid, acting as an AhR antagonist, counteracts this effect (Takei and Shimizu, 2026). In HCC, combining a TDO inhibitor with irinotecan showed enhanced anti-tumor efficacy via AhR pathway modulation (Liu et al., 2021). These cross-cancer data, though preclinical, support the rationale for AhR-based combination strategies beyond CRC (Table 3). Furthermore, the nanobody VHH212 targeting hypoxia-inducible factor-1α (HIF-1α), although not directly targeting AhR, suppresses the HIF-1/VEGF pathway and achieved an 80.44% tumor inhibition rate in preclinical xenograft models of pancreatic cancer when combined with gemcitabine (Kang et al., 2021). However, this result was obtained in a single animal model and should be interpreted with caution, as xenograft models do not recapitulate the immune microenvironment and stromal complexity of human PDAC. This finding provides indirect, preclinical evidence for targeting AhR-downstream or related pathways, but clinical validation is warranted (Figure 4).

Beyond pharmacological AhR modulators, microbiome-targeted interventions represent an emerging and mechanistically grounded adjunctive strategy for AhR-directed cancer therapy. Three complementary approaches merit consideration. First, probiotic supplementation with defined AhR ligand-producing strains—such as *Lactobacillus* reuteri (I3A producer), *Lactobacillus* paracasei ZJUZ2-3 (IAA producer) (Yang et al., 2025), and Bifidobacterium breve (ILA producer) (Li Y. et al., 2024)—can selectively deliver beneficial AhR agonists to the tumor microenvironment. However, strain selection requires caution, as L. murinus exacerbates PDAC through immunosuppressive AhR activation in macrophages (Hezaveh et al., 2022), illustrating that probiotic effects are context- and strain-dependent. Second, dietary interventions offer a scalable approach to modulate AhR ligand availability. Tryptophan-enriched diets potentiate I3A-mediated AhR activation in CD8^+^ T cells and enhance ICI response (Bender et al., 2023), while cruciferous vegetable-derived I3C and its dimer DIM activate AhR to suppress tumor growth (Liu et al., 2021). Conversely, tryptophan restriction has shown efficacy in MYC-driven liver cancer models by depleting kynurenine-mediated AhR activation (Ternes et al., 2022), highlighting the context-dependent rationale for either enriching or restricting dietary tryptophan based on tumor type and stage. Third, fecal microbiota transplantation (FMT) can reshape the entire microbial AhR ligand landscape. The RENMIN-215 phase II trial demonstrated that FMT combined with tislelizumab and fruquintinib achieved a disease control rate of 95% in refractory microsatellite-stable metastatic CRC (Zhao et al., 2023), providing the first clinical evidence that microbiome modulation can overcome immunotherapy resistance in gastrointestinal cancer. Mechanistically, FMT may restore beneficial indole-producing commensals while displacing kynurenine-generating pathogens, thereby rebalancing AhR signaling from pro-tumorigenic to tumor-suppressive. Collectively, these microbiome-targeted strategies—probiotics, dietary modulation, and FMT—constitute a fourth pillar of AhR-directed combination therapy, complementing pharmacological antagonists, SAhRMs, and PROTACs.

## 6 Application and challenges of preclinical models in AhR-targeted drug development

### 6.1 Genetically engineered mouse models and organoid models

Genetically engineered mouse models and patient-derived organoids (PDOs) are essential tools for dissecting the spatiotemporal specificity of AhR function and for evaluating targeted therapies. As detailed in Section 3.1, intestinal epithelium-specific AhR knockout mouse models have shown that AhR deficiency promotes the formation of colorectal precancerous lesions (Garcia-Villatoro et al., 2020), and a high-fat diet synergizes with AhR deficiency to promote colorectal cancer progression. In Apc/Kras mutant mice, intestinal epithelial AhR knockout promotes colonic stem/progenitor cell expansion by upregulating Wnt signaling, thereby exacerbating tumorigenesis (Han et al., 2021). In an inflammation-driven colorectal cancer model, AhR deficiency similarly enhances tumor growth, and its anti-tumor activity depends on the suppression of FOXM1 signaling (Safe et al., 2023). These models indicate that, at early stages of colon carcinogenesis, AhR exerts tumor-suppressive functions through inhibition of the Wnt/FOXM1 axis. However, genetically engineered mouse models are limited in their ability to recapitulate the full landscape of human tumors, particularly with respect to the heterogeneity and microenvironmental complexity that characterize human gastrointestinal malignancies. Therefore, findings derived from these models still require complementary validation in human-derived systems and patient datasets.

PDOs retain the genetic characteristics of the original tumor and can be used for high-throughput screening and personalized evaluation of AhR-targeted therapies (Choi et al., 2025). Pyrazolopyrimidine derivatives (e.g., compound 7k) have demonstrated AhR antagonistic activity in zebrafish and syngeneic colorectal cancer models, selectively inhibiting cancer cells, downregulating AhR-related genes and PD-1/PD-L1 expression, and enhancing anti-tumor immunity when combined with anti-PD-1 therapy. PHGDH is highly expressed in colorectal cancer and positively correlates with AhR; knockdown of PHGDH reduces AhR activity and increases sensitivity to the selective AhR antagonist SR1 (Li HM. et al., 2024). Combining PDOs with molecular subtyping can identify sensitive tumor subpopulations, thereby guiding precision therapy. The integrated use of genetically engineered mouse models and PDOs can advance AhR-targeted therapy development at both the systemic *in vivo* level and the individualized *in vitro* level. However, although PDOs preserve patient-specific genomic features, they lack key components of the tumor microenvironment, including immune cells, cancer-associated fibroblasts, and vascular structures, which precludes accurate assessment of AhR-mediated immunomodulation.

### 6.2 Microbiome–AhR interaction models

Studying the interaction between the microbiome and AhR is crucial for understanding gastrointestinal tumor mechanisms. Germ-free mice and fecal microbiota transplantation (FMT) models are key tools for exploring this network. These models allow separation of host genetic background from microbial influences, enabling direct validation of the functions of specific microbial communities or their metabolites. FMT studies have shown that modulation of the gut microbiota affects tryptophan metabolism and consequently regulates AhR activity (Wrzosek et al., 2021). In gastrointestinal tumors, *Lactobacillus* paracasei ZJUZ2-3 isolated from healthy gastric tissue activates AhR by secreting indole-3-acetic acid (IAA), competitively binds to MTDH to block NF-κB signaling, and suppresses tumor growth (Yang et al., 2025).

As noted in Section 5.4, the RENMIN-215 phase II trial demonstrated that FMT combined with tislelizumab and fruquintinib achieved a 95% disease control rate in refractory MSS metastatic CRC, with responders exhibiting enrichment of Proteobacteria and Lachnospiraceae (Zhao et al., 2023). While this trial was not designed to specifically quantify AhR pathway modulation, the observed efficacy is consistent with FMT-mediated restoration of beneficial indole-producing commensals and rebalancing of tryptophan metabolism. In melanoma, FMT from anti-PD-1 responders has been shown to restore immunotherapy efficacy in non-responders (Routy et al., 2023). These clinical data, while primarily obtained in non-GI malignancies, provide a proof-of-concept that microbiome manipulation can modulate AhR-relevant immune pathways and justify the design of FMT-AhR biomarker-integrated trials in gastrointestinal cancers. Germ-free mouse models confirmed that live bacterial injection is effective, whereas heat-killed bacteria are not. Nevertheless, FMT models are constrained by considerable inter-donor variability and inconsistent engraftment efficiency of transferred microbial consortia. Moreover, the immature immune systems of germ-free recipients may fundamentally confound the interpretation of AhR-dependent signaling. These limitations collectively underscore the need for caution when extrapolating findings from microbiome–AhR models to human disease contexts.

## 7 Current challenges and limitations in AhR-targeted drug development

### 7.1 Complexity and duality of AhR signaling

The central challenge in AhR-targeted therapy stems from the context-dependent transition between its “gatekeeper” and “promoter” modes (defined in the Introduction). The same receptor can preserve barrier integrity and suppress tumorigenesis in one context while driving immune evasion and metabolic reprogramming in another. This functional duality—governed by ligand identity, disease stage, cell type, and microenvironmental composition—means that its functions can be diametrically opposed across different conditions, posing a major challenge for targeted therapy. Within tumor cells, AhR can either suppress or promote carcinogenesis. For example, in colorectal cancer, intestinal epithelium-specific knockout of AhR upregulates Wnt signaling and thereby promotes expansion of Apc/Kras mutation-driven tumor stem/progenitor cells (Han et al., 2021). In contrast, in EBV-associated gastric cancer, the viral protein LMP2A suppresses AhR expression and activity, limiting its pro-proliferative function (Jiang et al., 2021).

AhR activation is typically associated with immunosuppression. Kynurenine promotes Treg differentiation and PD-L1 upregulation through AhR, mediating immune evasion and liver metastasis in colorectal cancer (Miyazaki et al., 2022); the traditional Chinese medicine formula Jianpi Jiedu decoction suppresses AhR and M2 macrophage polarization by modulating the gut microbiota (Chang et al., 2024). However, in colitis-associated colorectal cancer, AhR activation suppresses NLRP3 inflammasomes and alleviates inflammation (Ngui et al., 2020). The environmental endocrine disruptor nonylphenol inhibits TL1A expression via the endothelial AhR/HDAC2/HNF4α pathway, promoting angiogenesis in colorectal cancer (Zhang T. et al., 2022). The *Fusobacterium* nucleatum metabolite FTIN stimulates proliferation through the AhR–TERT axis, whereas the probiotic Akkermansia muciniphila suppresses the AhR/β-catenin pathway to inhibit colorectal cancer (Zhang L. et al., 2023).

Notably, AhR expression level alone is insufficient to predict therapeutic efficacy, as its function is influenced by ligand type, co-activators, and cross-talk with pathways such as Wnt and STAT3 (Yan et al., 2021; Lee et al., 2022). Moreover, the expression patterns of CYP1A1/1B1 differ between tumor cells and stromal cells (Perrot-Applanat et al., 2024); and the clinical significance of the AhR-interacting protein AIP, although associated with gastric cancer progression, remains unclear for predicting treatment response (Díaz Del Arco et al., 2020). Future efforts should integrate genomic, metabolomic, and microbiomic data to develop precise biomarker panels, enabling personalized application of AhR-targeted therapies.

### 7.2 Ligand specificity and off-target toxicity

The pleiotropy of AhR ligands and their off-target toxicity represent central challenges in drug development. Many AhR ligands lack high specificity and may simultaneously affect other nuclear receptors or signaling pathways. Although potent agonists such as TCDD activate AhR, prolonged exposure can lead to severe toxicities, including immunosuppression and teratogenicity (Zhang W. et al., 2022). AhR activation can also modulate a positive feedback loop involving gut microbial metabolites (e.g., formate), thereby exacerbating tumor progression (Ternes et al., 2022). Therefore, rigorous toxicological evaluation of AhR ligands—particularly with respect to immunotoxicity and carcinogenic risk under long-term, low-dose exposure—is essential for clinical safety.

Developing AhR ligands with high selectivity, favorable pharmacokinetic profiles, and appropriate tissue distribution remains a major challenge in chemical synthesis. Ideal ligands must achieve effective concentrations at the tumor site while minimizing exposure to normal tissues. Gut microbiota-derived indole-3-acetic acid can inhibit colorectal cancer proliferation through AhR-independent pathways (e.g., TLR4–JNK), offering a potential alternative that bypasses classical toxic pathways (Tomii et al., 2023). This observation has catalyzed interest in postbiotics—defined preparations of inanimate microorganisms and/or their metabolites that confer health benefits (Salminen et al., 2021). Purified AhR-active microbial metabolites, including ILA, I3A, and IPA, offer several advantages over live probiotics: defined chemical composition, controllable dosing, reduced infection risk in immunocompromised patients, and circumvention of colonization variability. However, postbiotic development faces distinct challenges: many indole metabolites have poor oral bioavailability, rapid systemic clearance, and limited tumor-site accumulation. Advanced delivery systems—including nanoparticle encapsulation and colon-targeted release formulations—are being explored to enhance bioavailability and tissue specificity (Xie et al., 2024). The transition from crude probiotic preparations to defined postbiotic compounds represents a critical step toward standardizing microbiome-targeted AhR modulation for clinical use. Ligand selectivity is also reflected in differential AhR activation across species or isoforms; for example, zebrafish and medaka exhibit orders-of-magnitude differences in sensitivity to TCDD (Zhang W. et al., 2023; Zhang et al., 2024). Computational toxicology and bioinformatics approaches are needed for early screening of safe compounds (Yang et al., 2026). Overcoming ligand pleiotropy and off-target toxicity will require multidisciplinary collaboration—from structural optimization to systematic toxicological evaluation—to advance AhR-targeted drugs toward clinical application.

## 8 Future perspectives and translational research directions

### 8.1 AhR functional stratification framework

To address the need for a more structured framework, we propose a three-dimensional AhR functional stratification model (Table 4) that categorizes AhR activity along three orthogonal axes:

- Ligand type: distinguishing persistent environmental agonists (e.g., TCDD, PAHs) that drive chronic, pro-tumorigenic AhR activation from transient dietary and endogenous ligands (e.g., I3C, kynurenine) whose effects are context-dependent (Murray et al., 2014; Peng et al., 2026; Opitz et al., 2011).
- Tumor stage: contrasting the tumor-suppressive functions of AhR during early inflammation-associated carcinogenesis—where AhR loss accelerates AOM/DSS-induced colorectal tumorigenesis and I3C supplementation reduces tumor multiplicity by 92% (Díaz-Díaz et al., 2016)—with its tumor-promoting role in advanced disease, where the TDO2-kynurenine-AhR axis drives PD-L1 transactivation, cancer stemness, and liver metastasis (Miyazaki et al., 2022; Opitz et al., 2011).
- Microenvironmental cues: encompassing immune infiltration status, microbiota composition, and metabolite landscapes that collectively determine whether AhR signaling supports or suppresses anti-tumor immunity (Hezaveh et al., 2022; Campesato et al., 2020; Cui et al., 2024).

**TABLE 4:** A structured functional stratification framework for aryl hydrocarbon receptor (AhR) in gastrointestinal cancers.

Stratification Dimension	Stratification Parameter	Representative Ligands/Conditions	Functional Outcome	Molecular Mechanism	GI cancer Context	Therapeutic Implication	Key References
Ligand Type	Environmental pollutants (TCDD, PAHs, PCBs) vs Dietary/endogenous ligands (I3C, DIM,Kyn, ITE, FICZ)	• Persistent agonists TCDD, B[a]P, PCBs • Transient agonists I3C, DIM, ICZ • Endogenous Kynurenine (Kyn) Kynurenic acid (KynA)	• Persistent activation → Pro-tumorigenic • Transient activation → Anti-tumorigenic (context-dependent)	• TCDD: sustained nuclear localization, chronic CYP1A1/1B1 induction, mutagenic metabolite generation • Kyn: activates AhR→ IDO1/TDO2 amplification loop, PD-1↑ on CD8^+^T cells • I3C/DIM: FOXM1↓ IL-22/STAT3 signaling, enhanced DNA repair	• Gastric cancer: H. pylori-derived PAHs activate AhR→promote invasion • CRC: Kyn-IDO1 axis drives β-catenin activation and immune evasion • HCC: TCDD-induced CYP1A1 promotes hepatocarcinogenesis	• AhR antagonists for IDO1/TDO2-high tumors • Dietary I3C/DIM as chemopreventive agents in IBD-associated CRC • Selective AhR modulators (SAhRMs) to dissociate anti-tumor from toxic effects	Murray et al. (2014), Peng et al. (2026), Opitz et al. (2011), Díaz-Díaz et al. (2016)
Tumor Stage	Early stage (inflammation/premalignant) vs. Late stage (metastasis /drug resistance)	• Early: dietary indoles (I3C), microbial tryptophan metabolites (IAA, IPA, IAld) • Late: tumor-derived Kyn (IDO1/TDO2↑), IL4I1-derived I3P	• Early stage: → Tumor-suppressive (anti-inflammatory, barrier maintenance) • Late stage: → Tumor-promoting (immune evasion, stemness,metastasis)	• Early: AhR→IL-22/STAT3→DNA repair;AhR→FOXM1↓→anti- proliferation; AhR→anti-inflammatory cytokine regulation • Late: TDO2-Kyn-AhR→ PD-L1 transactivation;Wnt/β-catenin →CSC maintenance; PI3K/Akt→ drug resistance	• CRC (early): AhR loss accelerates AOM/DSS- induced tumorigenesis; I3C reduces tumors 92% • CRC (late): TDO2-AhR →liver metastasis, 5-FU/oxaliplatin resistance • PDAC: Kyn-AhR in TAMs → immune exclusion, checkpoint resistance	• Early-stage intervention: AhR agonists (I3C/DIM) for chemoprevention in high-risk IBD patients • Late-stage intervention: AhR antagonists + anti- PD-1/PD-L1 combination for metastatic disease • Stage-specific clinical trial design warranted	Hezaveh et al. (2022), Miyazaki et al. (2022), Opitz et al. (2011), Díaz-Díaz et al. (2016)
Microenvironment Cues	Immune infiltration Status/Microbiota Composition/Metabolitelandscape	• Immune-excluded/desert TME: Kyn↑, Treg↑, TAM (M2)↑ • Immune-inflamed TME: CD8^+^ T cell↑, IFNγ↑, AhR activity↓ • Microbiota: *Lactobacillus* (indole+), *Fusobacterium* (Kyn shift)	• Immunosuppressive TME → Tumor progression, checkpoint resistance • Immunoactive TME → Tumor control Checkpoint sensitivity	• Kyn-AhR→Treg/TAM suppressive axis; CD39↑, IL-10↑, Arg1↑ • Microbial indoles→ TAM AhR activation→ CD8^+^ T cell suppression • IDA-AhR-ALDH1A3→ ferroptosis resistance • FICZ/IAA→Th17/IL-22→ mucosal barrier repair	• PDAC: Lactobacillus- derived indoles→TAM AhR→immune suppression; dietary Trp restriction restores T cell function • CRC: Peptostreptococcus- derived IDA→ferroptosis resistance; Akkermansia→ AhR/Wnt axis inhibition • Gastric: H. pylori-microbiota shift alters AhR ligand availability	• AhR inhibitors (e.g., CH-223191) + anti-PD-1 for immune-excluded tumors (IDO1/TDO2-high) • Microbiota modulation (FMT, dietary Trp adjustment) to reshape AhR ligand landscape • Patient stratification by tissue AhR signature/serum Kyn/Trp ratio	Hezaveh et al. (2022), Han et al. (2021), Campesato et al. (2020), Miyazaki et al. (2022), Cui et al. (2024)

This table presents a three-dimensional functional stratification framework for the aryl hydrocarbon receptor (AhR) in gastrointestinal (GI) cancers. The framework integrates evidence from across the manuscript to define stratification criteria along three axes—ligand type, tumor stage, and microenvironmental cues—each with distinct functional outcomes, molecular mechanisms, GI, cancer-specific contexts, and therapeutic implications. Abbreviations: AhR, aryl hydrocarbon receptor; AOM, azoxymethane; CRC, colorectal cancer; CSC, cancer stem cell; DSS, dextran sulfate sodium; FICZ, 6-formylindolo[3,2-b][3,2-b]carbazole; FMT, fecal microbiota transplantation; HCC, hepatocellular carcinoma; I3C, indole-3-carbinol; IAA, indole-3-acetic acid; IAld, indole-3-aldehyde; IDA, trans-3-indoleacrylic acid; IDO1, indoleamine 2,3-dioxygenase 1; IPA, indole-3-propionic acid; ITE, 2-(1H-indol-3-ylcarbonyl)thiazole-4-carboxylic acid; Kyn, kynurenine; PAHs, polycyclic aromatic hydrocarbons; PCBs, polychlorinated biphenyls; PDAC, pancreatic ductal adenocarcinoma; SAhRMs, selective AhR modulators; TAM, tumor-associated macrophage; TCDD, 2,3,7,8-tetrachlorodibenzo-p-dioxin; TDO2, tryptophan 2,3-dioxygenase 2; TME, tumor microenvironment; Trp, tryptophan.

This framework has direct translational implications: early-stage, inflammation-driven lesions may benefit from AhR agonist-based chemoprevention, whereas advanced, immune-excluded tumors with active IDO/TDO-kynurenine-AhR signaling are candidates for AhR antagonist therapy in combination with immune checkpoint blockade (Campesato et al., 2020; Miyazaki et al., 2022). Critically, patient stratification by tissue AhR signature, serum kynurenine/tryptophan ratio, and microbiome-derived indole profiles could guide stage- and context-specific therapeutic interventions—AhR agonists for early-stage chemoprevention, AhR antagonists for advanced immune-excluded tumors, and microbiota modulation for reshaping the AhR ligand landscape.

### 8.2 Development of precision diagnostic tools for AhR signaling

The activity status of the AhR signaling pathway varies considerably among cell types and microenvironments in gastrointestinal tumors (Haarmann-Stemmann et al., 2025); therefore, developing diagnostic tools to precisely assess AhR activity is crucial for patient stratification and targeted therapy. Multi-omics technologies provide robust support for this goal. Transcriptomics can delineate the expression profiles of AhR downstream target genes (e.g., CYP1A1, CYP1B1) and construct molecular signatures (Goya-Jorge et al., 2020). In colorectal cancer, AhR expression is positively correlated with PHGDH, and coordinated changes in their target genes may serve as potential biomarkers (Li HM. et al., 2024). Proteomics can verify the expression and translocation of AhR and its chaperone proteins (e.g., HSP90, XAP2, p23) (Ue et al., 2020). Metabolomics detects levels of endogenous ligands such as kynurenine and indole-3-propionic acid (Park et al., 2020); notably, 3-indolepropionic acid derived from *Fusobacterium* nucleatum promotes M2 polarization and immunosuppression by activating macrophage AhR (Song et al., 2025).

Integration of multi-omics data can construct a multidimensional AhR activity signature that distinguishes its pro-oncogenic versus tumor-suppressive functions (Safe et al., 2023), thereby enabling the development of molecular diagnostic kits. In esophageal cancer, high AhR expression correlates with disease progression, and omeprazole inhibits cancer cells via the AhR pathway (Bai et al., 2021). Precision diagnostics can identify patient subgroups with aberrant AhR activation or loss of function (Dvořák et al., 2025). In colorectal cancer, the efficacy of AhR antagonists (e.g., compound 7k) depends on AhR activity within the tumor microenvironment (Choi et al., 2025). In summary, developing precision diagnostic tools is a cornerstone for personalized AhR-targeted therapy, as it improves treatment outcomes, reduces adverse effects, and paves a new path for precision medicine in gastrointestinal cancers.

### 8.3 Exploration of novel AhR regulatory mechanisms and drug targets

The functional regulation of AhR involves post-translational modifications, non-coding RNAs, and chromatin interactions, offering opportunities for identifying new druggable nodes. Phosphorylation and ubiquitination regulate AhR stability and activity: in the unliganded state, AhR binds to HSP90, p23, and XAP2 (Ue et al., 2020); ligand binding induces conformational changes and nuclear translocation, whereas ubiquitination determines its degradation rate (Sondermann et al., 2023). Non-coding RNAs can target AhR expression; for example, microRNAs in colorectal cancer influence CYP1A1/CYP1B1 expression (Li HM. et al., 2024). AhR also interacts with chromatin remodeling complexes, restricting chromatin accessibility at Yap/Tead target genes to prevent excessive proliferation (Shah et al., 2022). Elucidating these mechanisms may lead to the discovery of entirely new drug targets.

Disrupting AhR–ARNT dimerization or AhR–cofactor interactions is a key focus of inhibitor development. Blocking formation of the AhR–ARNT dimer inhibits transcriptional activity, and computer-aided drug design is accelerating the discovery of such inhibitors (Dvořák et al., 2025). In colorectal cancer, AhR suppresses FOXM1 to exert anti-tumor effects; therefore, enhancing AhR binding to tumor-suppressive cofactors or blocking its interaction with pro-oncogenic cofactors could enable precise regulation (Safe et al., 2023). Regarding the non-canonical pathway, the *Fusobacterium* nucleatum metabolite 3-indolepropionic acid promotes M2 polarization and colorectal cancer progression by activating macrophage AhR (Song et al., 2025); conversely, pyrazolopyrimidine derivatives (e.g., compound 7k), acting as AhR antagonists, synergistically enhance anti-PD-1 immune responses in colorectal cancer models (Choi et al., 2025). Rational design of SAhRMs holds theoretical promise in preclinical settings for overcoming non-selective side effects and achieving precision therapy (Safe et al., 2020); however, this promise has not yet been realized clinically, as no SAhRM has advanced to oncology clinical trials. The translational gap between structural optimization and clinical efficacy remains a significant challenge.

### 8.4 Advancing AhR-based combination therapies into the clinic

Combining AhR-targeted drugs (particularly SAhRMs and antagonists) with immunotherapy, chemotherapy, or radiotherapy represents a rational but preclinically unvalidated strategy for promoting clinical translation, aiming to overcome the limitations of monotherapy through multi-pathway synergy. However, the clinical evidence supporting such combinations remains limited: as detailed in Section 4.1, the Phase I trial of BAY 2416964 demonstrated manageable safety but only modest single-agent activity, and no combination regimen with AhR antagonists has reported Phase II efficacy data in gastrointestinal cancers to date. Sorafenib, a multi-kinase inhibitor, has been shown to act as an AhR antagonist (Wei et al., 2022), and its combination with AhR agonists or immune checkpoint inhibitors warrants further exploration. Etoricoxib enhances AhR activity (Fang et al., 2023), providing a rationale for its combination with AhR-targeted agents in inflammatory gastrointestinal tumors. Given that AhR exerts both anti-tumor and pro-tumor effects within the tumor microenvironment (Rejano-Gordillo et al., 2022), well-designed phase I/II clinical trials are needed to evaluate the safety, tolerability, and anti-tumor activity of combination regimens, while rigorously monitoring specific toxicities and optimizing dosing schedules.

Biomarker-driven patient enrichment is the cornerstone for improving the success rate of clinical trials. Potential biomarkers include AhR and CYP1A1 expression levels, tryptophan metabolite concentrations, features of the immune microenvironment, and—critically—microbiome composition metrics such as the abundance of AhR ligand-producing commensals (e.g., *Lactobacillus*, Bifidobacterium, Akkermansia) versus kynurenine-generating pathogens. Integrating stool metagenomic profiling with serum metabolomics (quantifying I3A, ILA, IPA, and kynurenine levels) could enable a composite “AhR activity index” that distinguishes patients likely to benefit from AhR agonist-based microbiome interventions (e.g., probiotic supplementation, tryptophan-enriched diet) from those requiring AhR antagonist strategies (e.g., IK-175, SAhRMs). This microbiome-informed stratification is particularly relevant for combination trials: patients with depleted beneficial indole-producing commensals may be prioritized for FMT or probiotic adjuncts alongside ICI, while those with elevated kynurenine/AhR signaling may benefit from AhR antagonist plus ICI combinations. AhR exerts anti-tumor effects in part by regulating IL-22 (Safe et al., 2023), suggesting that IL-22-related molecules could serve as additional biomarkers. AhR engages in cross-talk with pathways such as HIF-1α, NF-κB, and STAT3 (Chong et al., 2023), and its activity status influences therapeutic responses. Multi-omics-based patient stratification can identify populations most likely to benefit, thereby enabling precision medicine and accelerating clinical translation.

## 9 Conclusion

In gastrointestinal tumors, AhR has evolved from an environmental toxin sensor into a context-dependent regulatory hub that oscillates between “gatekeeper” and “promoter” modes. This functional duality—encompassing both homeostatic/anti-oncogenic and pro-oncogenic roles—arises from ligand diversity, signaling complexity, and tissue and cell-type specificity. Simply classifying AhR as “oncogenic” or “anti-oncogenic” is no longer sufficient; rather, future research must precisely map the dynamic gatekeeper-to-promoter transition across specific tumor types, disease stages, and microenvironmental cellular compartments. Accordingly, we propose shifting the therapeutic paradigm from a binary classification of AhR as “good” or “bad” to a dynamic, context-informed decision matrix that identifies whether AhR is operating in its “gatekeeper” or “promoter” mode in a given patient and selects interventions accordingly—agonists to reinforce gatekeeper function in early-stage, inflammation-driven lesions; antagonists to block promoter activity in advanced, immune-excluded tumors; and microbiota modulation to reshape the ligand landscape.

AhR-directed drug development is aspirationally transitioning from a “blockade” paradigm toward “precision modulation,” as conceptually exemplified by SAhRMs and PROTAC-based degraders. However, these emerging modalities remain at an early preclinical stage, and no AhR-targeted agent has yet demonstrated robust single-agent efficacy in gastrointestinal cancers in clinical settings. As detailed in Section 4.1, early clinical trials (BAY 2416964, IK-175) have confirmed target engagement and immune modulation but yielded only modest clinical activity, underscoring the substantial gap between preclinical promise and clinical reality. These early data caution against extrapolating preclinical efficacy directly to the clinic and highlight the critical need for biomarker-driven patient stratification.

Targeting AhR in gastrointestinal cancers remains a scientifically compelling but as-yet unproven therapeutic strategy. Several context-specific risks must be acknowledged. First, the same AhR-targeted agent may produce diametrically opposite outcomes depending on tumor stage, ligand milieu, and immune context, meaning that timing and patient selection are as critical as drug design itself. Second, complete AhR blockade risks disrupting its physiological functions in maintaining intestinal barrier integrity, immune homeostasis, and hepatic metabolic balance, potentially exacerbating inflammation or metabolic disturbances (Griffith and Frankel, 2024). Third, increasing the dose of AhR inhibitors does not necessarily improve anti-tumor outcomes in preclinical models, likely because a balance between beneficial and detrimental AhR signaling is reached at a certain threshold—a phenomenon that complicates dose optimization (Polonio et al., 2025). Fourth, the failure of the ECHO-301 Phase III trial for the upstream IDO1 inhibitor epacadostat (discussed in Section 4.1) serves as a cautionary precedent for the entire kynurenine–AhR axis (Muller et al., 2019).

Success in translating AhR-targeted therapies to the clinic will depend on overcoming these challenges through deep integration of basic and clinical research, rigorous biomarker-driven trial design, and multidisciplinary collaboration. A systematic elucidation of the context-dependent regulatory networks governed by AhR, coupled with the development of validated companion diagnostics, is a necessary—though not sufficient—step toward realizing the potential of AhR as a precision therapeutic target in gastrointestinal oncology. Until such evidence is generated, AhR-directed strategies should be regarded as promising but experimental approaches that require prospective validation in well-designed clinical trials.
